# Publication bias examined in meta-analyses from psychology and medicine: A meta-meta-analysis

**DOI:** 10.1371/journal.pone.0215052

**Published:** 2019-04-12

**Authors:** Robbie C. M. van Aert, Jelte M. Wicherts, Marcel A. L. M. van Assen

**Affiliations:** 1 Department of Methodology and Statistics, Tilburg University, Tilburg, the Netherlands; 2 Department of Sociology, Utrecht University, Utrecht, the Netherlands; University of Edinburgh, UNITED KINGDOM

## Abstract

Publication bias is a substantial problem for the credibility of research in general and of meta-analyses in particular, as it yields overestimated effects and may suggest the existence of non-existing effects. Although there is consensus that publication bias exists, how strongly it affects different scientific literatures is currently less well-known. We examined evidence of publication bias in a large-scale data set of primary studies that were included in 83 meta-analyses published in Psychological Bulletin (representing meta-analyses from psychology) and 499 systematic reviews from the Cochrane Database of Systematic Reviews (CDSR; representing meta-analyses from medicine). Publication bias was assessed on all homogeneous subsets (3.8% of all subsets of meta-analyses published in Psychological Bulletin) of primary studies included in meta-analyses, because publication bias methods do not have good statistical properties if the true effect size is heterogeneous. Publication bias tests did not reveal evidence for bias in the homogeneous subsets. Overestimation was minimal but statistically significant, providing evidence of publication bias that appeared to be similar in both fields. However, a Monte-Carlo simulation study revealed that the creation of homogeneous subsets resulted in challenging conditions for publication bias methods since the number of effect sizes in a subset was rather small (median number of effect sizes equaled 6). Our findings are in line with, in its most extreme case, publication bias ranging from no bias until only 5% statistically nonsignificant effect sizes being published. These and other findings, in combination with the small percentages of statistically significant primary effect sizes (28.9% and 18.9% for subsets published in Psychological Bulletin and CDSR), led to the conclusion that evidence for publication bias in the studied homogeneous subsets is weak, but suggestive of mild publication bias in both psychology and medicine.

## Introduction

Meta-analysis is the standard technique for synthesizing different studies on the same topic, and is defined as “the statistical analysis of a large collection of analysis results from individual studies for the purpose of integrating the findings” [1]. One of the greatest threats to the validity of meta-analytic results is publication bias, meaning that the publication of studies depends on the direction and statistical significance of the results [2]. Publication bias generally leads to effect sizes being overestimated and the dissemination of false-positive results (e.g., [3, 4]). Hence, publication bias results in false impressions about the magnitude and existence of an effect [5] and is considered one of the key problems in contemporary science [6].

Indications for the presence of publication bias are present in various research fields. The main hypothesis tested in the psychology and psychiatry literature is statistically significant in approximately 90% of the cases[7, 8], which is not in line with the on average low statistical power of about 50% or less in, for instance, psychology [9, 10] and may be caused by publication bias. Franco, Malhotra, and Simonovits [11] examined publication bias in studies that received a grant within the social sciences and found that 64.6% of the studies where most or all results did not support the alternative hypotheses was not written up compared to 4.4% of the studies where most or all the alternative hypotheses were supported (cf. [12, 13]). In a highly similar project within the psychological literature, Franco, Malhotra, and Simonovits [14] showed that 70% of the included outcomes in a study were not reported, and that this selective reporting depended on statistical significance of the outcomes. Although these findings suggest that publication bias is present in numerous research fields, mixed results were observed when analyzing the distribution of *p-*values [15–21] where a difference between *p*-values just above and below α = .05 may be interpreted as evidence for publication bias.

Compared to the social sciences, more attention has been paid to publication bias in medicine [22]. Medicine has a longer history in registering clinical trials before conducting the research (e.g., [23, 24]). As of 2007, the US Food and Drug Administration Act (FDA) even requires US researchers to make the results of different types of clinical trials publicly available independent of whether the results have been published or not [25]. With registers like *clinicaltrials*.*gov*, it is easier for meta-analysts to search for unpublished research, and to include it in their meta-analysis. Furthermore, it is straightforward to study publication bias by comparing the reported results in registers with the reported results in publications. Studies comparing the reported results in registers and publications show that statistically significant outcomes are more likely to be reported, and clinical trials with statistically significant results have a higher probability of getting published [26–28].

A number of methods exist to test for publication bias in a meta-analysis and to estimate a meta-analytic effect size corrected for publication bias. However, publication bias is often not routinely assessed in meta-analyses [29–31] or analyzed with suboptimal methods that lack statistical power to detect it [32, 33]. It has been suggested to reexamine publication bias in published meta-analyses [30, 34] by applying recently developed methods to better understand the severity and prevalence of publication bias in different fields. These novel methods have better statistical properties than existing publication bias tests and methods developed earlier to correct effect sizes for publication bias. Moreover, several authors have recommended to not rely on a single method for examining publication bias in a meta-analysis, but rather to use and report a set of different publication bias methods [35, 36]. This so-called triangulation should take into account that some methods do not perform well in some conditions and that none of the publication bias methods outperforms all the other methods under each and every condition; one method can signal publication bias in a meta-analysis whereas another one does not. Using a set of methods to assess the prevalence and severity of publication bias may yield a more balanced conclusion.

We set out to answer three research questions in this paper. The first research question concerned the prevalence of publication bias: “What is the prevalence of publication bias within published meta-analyses in psychological and medical research?” (1a), and “Is publication bias more prevalent in psychology than in medicine after controlling for the number of studies in a meta-analysis?” (1b). Medicine was selected to be compared to psychology, because more attention has been paid to publication bias in general [22] and study registration in particular (e.g., [23, 24]) within medicine. We also evaluated the amount of agreement between different publication bias methods. In the second research question, we examined whether effect size estimates of traditional meta-analysis and corrected for publication bias by the *p-*uniform method can be predicted by characteristics of a meta-analysis: “What are predictors of the meta-analytic estimates of traditional meta-analysis and *p*-uniform?”. Our third research question also consisted of two parts and is about overestimation of effect size caused by publication bias: “How much is effect size overestimated by publication bias in meta-analyses in psychology and medical research?” (3a), and “What are predictors of the overestimation in effect size caused by publication bias in meta-analyses in psychology and medical research?” (3b). The aim of this paper is to shed light on the prevalence of publication bias and the overestimation that it causes by answering the above stated research questions. As we focus on homogeneous (subsets of) meta-analyses (i.e., with no or small heterogeneity), we examine these questions for the population of homogeneous subsets. A large-scale dataset will be used containing 83 meta-analyses published in the psychological literature and 499 systematic reviews in the medical literature making this paper a thorough and extensive assessment of publication bias in psychological and medical research.

The hypotheses as well as our planned analyses were preregistered (see https://osf.io/8y5ep/) meaning that hypotheses, analysis plan, and code of the data analyses were specified in detail before the data were analyzed. Some additional analyses were conducted that were not included in the pre-analysis plan. We will explicate which analyses were exploratory when describing these analyses and their results. The paper continues by providing an overview of publication bias methods. Next, we describe the criteria for a meta-analysis to be included in our study. Then we describe how the data of meta-analyses were extracted and analyzed, and list our hypotheses. Subsequently, we provide the results of our analyses and conclude with a discussion.

## Publication bias methods

Methods for examining publication bias can be divided into two groups: methods that assess or test the presence of publication bias, and methods that estimate effect sizes corrected for publication bias. Methods that correct effect sizes for publication bias usually also provide a confidence interval and test the null hypothesis of no effect corrected for publication bias. Table 1 summarizes the methods together with their characteristics and recommendations on when to use each method. The last column of the table lists whether the method is included in our analyses. Readers that are not interested in the details regarding the publication bias methods can focus on the summary in Table 1.

**Table 1:** Summary of publication bias methods to assess publication bias and estimate effect sizes corrected for publication bias. The penultimate column lists principal references of the different methods and the final column indicates whether a method is included in the analyses of this paper.

	Method	Description	Characteristics/Recommendations	Included in analyses
**Assessing publication bias**				
	Fail-safe *N*	Estimates number of effect sizes in the file-drawer	Method is discouraged to be used, because it, for instance, assumes that all nonsignificant effect sizes are equal to zero and focuses on statistical instead of practical significance [39, 40].	No
	Funnel plot	Graphical representation of small-study effects where funnel plot asymmetry is an indicator of small-study effects	Publication bias is not the only cause of funnel plot asymmetry [43]. Eyeballing a funnel plot for asymmetry is subjective [46], so recommendation is to use a statistical test (i.e., Egger’s [43] or rank-correlation test [47]).	No
	Egger’s and rank-correlation test	Statistical tests for testing funnel plot symmetry	Publication bias is not the only cause of funnel plot asymmetry [43]. Methods are recommended to be applied when there are 10 or more effect sizes [48] otherwise the methods have low statistical power [47, 49].	Yes
	Test of Excess Significance	Computes whether observed and expected number of statistically significant results are in agreement	Do not apply the method in case of heterogeneity in true effect size [50]. Method is known to be conservative [51].	Yes
	*p-*uniform’s publication bias test	Examines whether statistically significant *p*-values are uniformly distributed at the estimate of the fixed-effect model	Method does not use information of nonsignificant effect sizes and, assumes homogeneous true effect size [5, 52].	Yes
**Correcting effect size for publication bias**				
	Trim and fill method	Method corrects for funnel plot asymmetry by trimming most extreme effect sizes and filling these effect sizes to obtain funnel plot symmetry	Method is discouraged to be used because it falsely imputes effect sizes when none are missing and other methods have shown to outperform trim and fill [5, 53, 54]. Moreover, funnel plot asymmetry is not only caused by publication bias [43], and the method does also not perform well if heterogeneity in true effect size is present [5, 55].	No
	PET-PEESE	Extension of Egger’s test where the corrected estimate is the intercept of a regression line fitted through the effect sizes in a funnel plot	Method becomes biased if it is based on less than 10 effect sizes, the between-study variance in true effect size is large, and the sample size of primary studies included in a meta-analysis is rather similar [56–59].	No
	*p*-uniform/*p*-curve	Estimate is the effect size for which the distribution of conditional *p*-values is uniformly distributed	Method does not use information of nonsignificant effect sizes and assumes homogeneous true effect size [5, 52, 53].	Yes
	Selection model approach	Method makes assumptions on the distribution of effect sizes (effect size model) and mechanism of observing effect sizes (selection model). Estimation is performed by combining these two models.	User has to make sophisticated assumptions and choices [39]. Large number of effect sizes (more than 100) are needed to avoid convergence problems [55, 60], but recent research showed that convergence problems of the approach by Iyengar and Greenhouse [61, 62] were only severe if there was no or extreme publication bias in combination with no or a small amount of heterogeneity in true effect size.	No
	10% most precise effect sizes	Only the 10% most precise effect sizes are used for estimation with a random-effects model	90% of the available effect sizes is discarded and bias in estimates increases as a function of heterogeneity in true effect size [63].	Yes

### Assessing or testing publication bias

The most often used method for assessing publication bias is fail-safe *N* [34, 37]. This method estimates how many effect sizes with a zero effect size have to be added to a meta-analysis for changing a statistically significant summary effect size in a meta-analysis to a nonsignificant result [38]. Applying the method is discouraged, because it makes the unrealistic assumption that all nonsignificant effect sizes are equal to zero, does not take study sample size into account, and focuses on statistical significance and not on the magnitude of an effect that is of substantial importance [39, 40].

**Fig 1:**
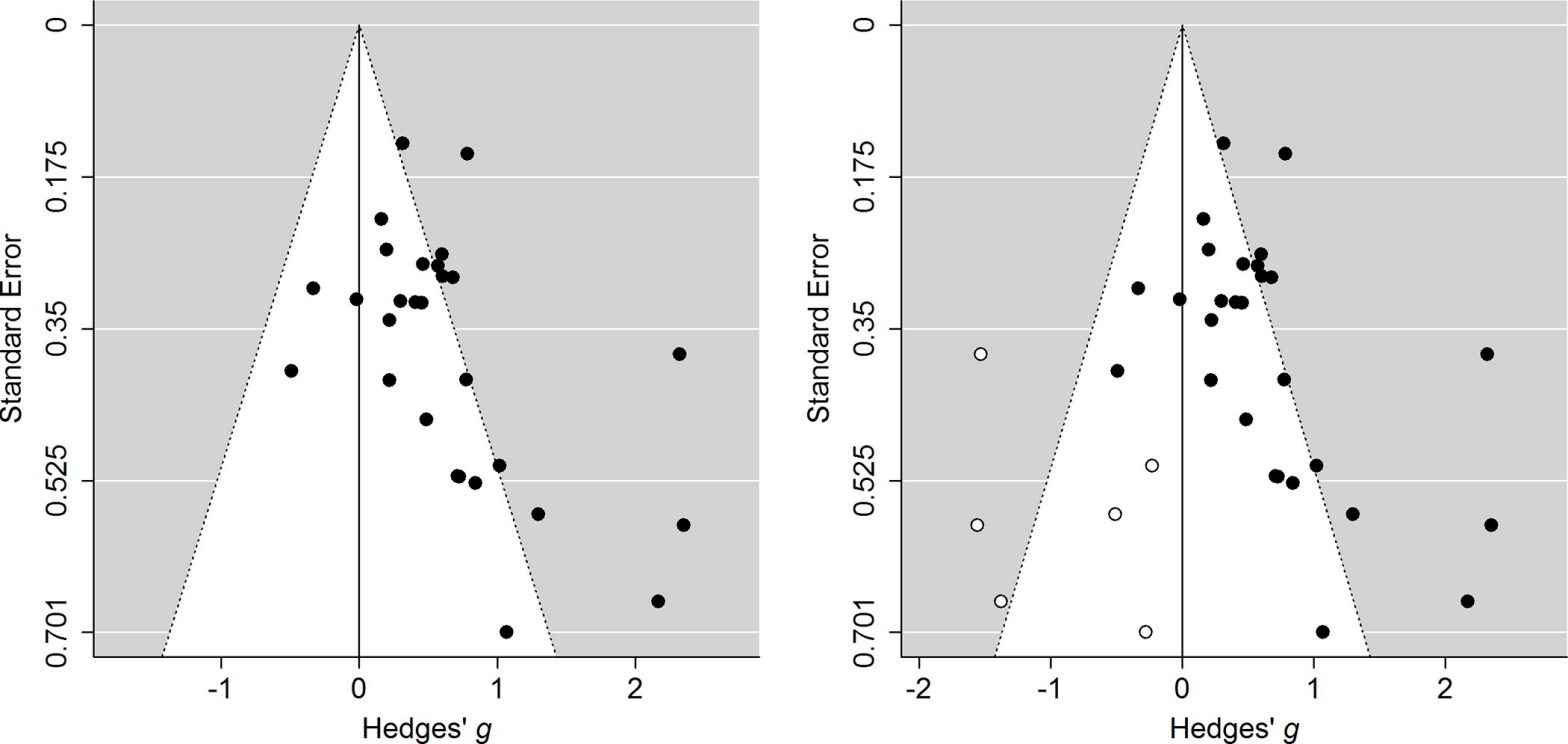
Funnel plot showing the relationship between the observed effect size (Hedges’ *g*; solid circles) and its standard error in a meta-analysis by Jürgens and Graudal [42] on the effect of sodium intake on Noradrenaline (left panel). The funnel plot in the right panel also includes the Hedges’ *g* effect sizes that are imputed by the trim and fill method (open circles).

Another popular method is the funnel plot [41]. In a funnel plot, the effect size estimates of the included studies in a meta-analysis are presented on the *x-*axis and some measure of the effect sizes’ precision is displayed on the *y-*axis. The left panel in Fig 1 shows a funnel plot for a meta-analysis in the systematic review by Jürgens and Graudal [42] studying the effect of sodium intake on different health outcomes. Solid circles in the funnel plot indicate studies’ Hedges’ *g* effect sizes (*y*-axis) and their standard errors (*x*-axis). A funnel plot illustrates whether small-study effects are present. That is, whether there is a relationship between effect size and its precision. The funnel plot should be symmetric and resemble an inverted funnel in the absence of small-study effects, whereas a gap in the funnel indicates that small-study effects exist. Publication bias is one of the causes of small-study effects [43], but funnel plot asymmetry is often interpreted as evidence for publication bias in a meta-analysis. Small-study effects can also be caused by, for instance, researchers basing their sample size on statistical power analyses in combination with heterogeneity in true effect size (see supplemental materials of [44] and [45]). In this case, larger true effect sizes are associated with studies using smaller sample sizes, resulting in funnel plot asymmetry.

Evaluating whether small-study effects exist by eyeballing a funnel plot is rather subjective [46]. Hence, Egger’s regression test [43] and the rank-correlation test [47] were developed to test whether small-study effects are present in a meta-analysis. Egger’s regression test uses linear regression with the observed effect sizes as dependent variable and a measure of primary studies’ precision as predictor. Evidence for small-study effects is obtained if the slope of this regression line is significantly different from zero. The rank-correlation test computes the rank correlation (Kendall’s τ) between the study’s effect sizes and their precision to test for small-study effects. Drawback of these two tests is that statistical power to detect publication bias is low especially if there are few effect sizes in a meta-analysis [47, 49]. Hence, these methods are recommended to be only applied to meta-analyses with ten or more effect sizes [48].

The test of excess significance (TES) compares the number of statistically significant effect sizes in a meta-analysis with the expected number of statistically significant effect sizes [50]. The expected number of statistically significant effect sizes is computed by summing the statistical power of each primary study in a meta-analysis. More statistically significant results than expected indicate that some effect sizes are (possibly because of publication bias) missing from the meta-analysis. Ioannidis and Trikalinos [50] recommend to not apply the method if heterogeneity in true effect size is present. Moreover, the TES is known to be conservative [5, 51].

Another more recently developed method for examining publication bias is the *p*-uniform method [5, 52]. This method is based on the statistical principle that the distribution of *p-*values at the true effect size is uniform. For example, the distribution of *p-*values under the null hypothesis is uniform. Since in the presence of publication bias not all statistically nonsignificant effect sizes get published, *p-*uniform discards nonsignificant effect sizes and computes *p-*values conditional on being statistically significant. These conditional *p*-values should be uniformly distributed at the (fixed-effect) meta-analytic effect size estimate based on the significant and nonsignificant effect sizes, and deviations from the uniform distribution signals publication bias. *P-*uniform’s publication bias test was compared to the TES in a Monte-Carlo simulation study [5], and statistical power of *p*-uniform was in general larger than the TES except for conditions with a true effect size of zero in combination with statistically nonsignificant studies included in a meta-analysis. This simulation study also showed that Type-I error rate of *p*-uniform’s publication bias test was too low if the true effect size was of medium size. Limitations of *p*-uniform’s publication bias test are that it assumes that the true effect size is homogeneous (which is not very common, see for instance [64–66]), and that the method may inefficiently use the available information by discarding statistically nonsignificant effect sizes in a meta-analysis.

### Correcting effect sizes for publication bias

Publication bias tests provide evidence about the presence of publication bias in a meta-analysis. However, statistical power of publication bias tests is often low in practice [54], because the number of effect sizes in a meta-analysis is often small. For instance, the median number of effect sizes in meta-analyses published in the Cochrane Database of Systematic Reviews was equal to 3 [67, 68]. Furthermore, the magnitude of the effect after correcting for publication bias is more of interest from the perspective of an applied researcher.

The most popular method to correct for publication bias in a meta-analysis is trim and fill [69, 70]. This method corrects for funnel plot asymmetry by trimming the most extreme effect sizes from one side of the funnel plot and filling these effect sizes in the other side of the funnel plot to obtain funnel plot symmetry. The corrected effect size estimate is obtained by computing the meta-analytic estimate based on the observed and imputed effect sizes. Trim and fill can also be used to create a confidence interval and test the null hypothesis of no effect after adjusting for funnel plot asymmetry. The procedure of trim and fill is illustrated in the right panel of Fig 1. The most extreme effect sizes from the right-hand side of the funnel plot are trimmed and imputed in the left-hand side of the funnel plot (open circles in the right panel of Fig 1). A drawback of trim and fill, which it shares with other methods based on the funnel plot, is that it corrects for small-study effects that are not necessarily caused by publication bias. Furthermore, the method cannot accurately correct for publication bias when the true effect size is heterogeneous (e.g., [5, 55]). Simulation studies have also shown that results of trim and fill cannot be trusted because it incorrectly adds studies when none are missing [55, 71, 72]. Hence, trim and fill is discouraged because of its misleading results [5, 53, 54].

The PET-PEESE method [65] is an extension of Egger’s regression test to estimate an effect size in a meta-analysis corrected for small-study effects. PET-PEESE is based on a regression analysis where the observed effect sizes are regressed on their standard errors by means of a weighted least squares regression with the inverse of the effect sizes’ sampling variances as weights. If the intercept is not significantly different from zero, the estimate of the intercept is interpreted as the effect size estimate corrected for publication bias. The estimate of the intercept reflects the effect size estimate in a study with a standard error of zero (i.e., a study with an infinite sample size). However, this estimator is biased if the intercept is significantly different from zero [65]. Hence, in case the intercept is significantly different from zero, the intercept of another weighted least squares regression analysis (with the inverse sampling variances as weights) is interpreted as the effect size estimate. In this regression analysis, the observed effect sizes are regressed on their sampling variances. Simulation studies have shown that PET-PEESE substantially reduced the overestimation caused by small-study effects [65]. However, PET-PEESE becomes biased when less than 10 effect sizes are included in a meta-analysis, the between-study variance in true effect size is large, and the sample size of primary studies included in a meta-analysis is rather similar [56–59].

The *p-*uniform method can also be used for estimating effect size (and a confidence interval) and testing the null hypothesis of no effect corrected for publication bias. *P-*uniform’s effect size estimate is equal to the effect size for which the *p-*values conditional on being statistically significant are uniformly distributed. A similar method that uses the distribution of conditional *p*-values for estimating effect size in the presence of publication bias is *p*-curve [53]. This method is similar to the *p*-uniform method, but differs in implementation (for a description of the difference between the two methods see [52]). A limitation of *p*-uniform and *p*-curve is that effect sizes are overestimated in the presence of heterogeneity in true effect size [52]. Especially if the heterogeneity in true effect size is more than moderate (*I*^2^ > 50%; more than half of the total variance in effect size is caused by heterogeneity) both methods overestimate the effect size, and their results should be interpreted as a sensitivity analysis. Another limitation of both methods is that they are not efficient if many nonsignificant effect sizes exist. Such results are discarded by the methods, yielding imprecise estimates and wide confidence intervals of *p-*uniform (*p-*curve does not estimate a confidence interval). *P-*uniform and *p-*curve both outperformed trim and fill in simulation studies [5, 53].

A selection model approach [45] can also be used for estimating effect size corrected for publication bias. A selection model makes assumptions on the distribution of effect sizes (i.e., effect size model) and the mechanism that determines which studies are selected (for publication) and hence observed (i.e., selection model). The effect size estimate (and confidence interval) corrected for publication bias is obtained by combining the effect size and selection model. Many different selection model approaches exist (e.g., [73–78]). Some approaches estimate the selection model [74, 77] whereas others assume a known selection model [79]. A recently proposed selection model approach [80] estimates effect size corrected for publication bias by using Bayesian model averaging over multiple selection models. Selection model approaches are hardly used in practice, because it requires sophisticated assumptions and choices [39] and a large number of effect sizes (more than 100) to avoid convergence problems [55, 60]. However, two recent simulation studies [61, 62] were conducted that included the three-parameter selection model approach by Iyengar and Greenhouse [74, 81] and showed that convergence problems of this approach were only severe for conditions that included only 10 studies, or conditions wherein publication bias was extreme.

Stanley, Jarrel, and Doucouliagos [63] proposed to correct for publication bias in the effect size estimate by computing the unweighted mean of the 10% most precise observed effect sizes, or the single most precise study in case of less than ten effect sizes. The rationale underlying only using the 10% most precise observed effect sizes is that these primary study’s effect sizes are less affected by publication bias than the 90% less precise discarded effect sizes. We propose to not combine the 10% most precise observed effect sizes with an unweighted mean, but with a random-effects model to take differences in primary study’s sampling variances and heterogeneity in true effect size into account. A disadvantage of this method is that it is not efficient leading to imprecise estimates and wider confidence intervals than estimation based on all effect sizes since up to 90% of the data is discarded. Moreover, bias in the method’s estimates increases as a function of the heterogeneity in true effect size [63].

## Methods

### Data

A large-scale data set was created with meta-analyses published between 2004 and 2014 in Psychological Bulletin and in the Cochrane Library to study the extent and prevalence of publication bias in psychology and medicine. Psychological Bulletin was selected to represent meta-analyses in psychology, because this journal publishes many meta-analyses on a variety of topics from psychology. Meta-analyses published in the Cochrane Database of Systematic Reviews (CDSR) of the Cochrane Library were used to represent medicine. This database is a collection of peer-reviewed systematic reviews conducted in the field of medicine.

A first requirement for the inclusion of a meta-analysis was that either fixed-effect or random-effects meta-analysis had to be used in the meta-analysis (i.e., no other meta-analytic methods as, for instance, meta-analytic structural equation modelling or multilevel meta-analysis). Another requirement was that sufficient information in the meta-analysis had to be available to compute the primary study’s standardized effect size and its sampling variance. The same effect size measure (e.g., correlation and standardized mean difference) as in the original meta-analysis was used to compute the primary study’s effect size and its sampling variance. Formulas as described in [82], [83], and [84] were used for computing the standardized effect sizes and their sampling variances. For each included primary study, we extracted information on effect size and sampling variance, as well as information on all categorical moderator variables. Based on these moderators, we created homogeneous subsets of effect sizes. That is, a homogeneous subset consisted of the effect sizes that had the same scores on all the extracted moderators. Consequently, each meta-analysis could contain more than one subset of effect sizes if multiple homogeneous subsets were extracted based on the included moderators.

We only included subsets with less than moderate heterogeneity (*I*^2^<50%) [85], because none of the publication bias methods has desirable statistical properties under extreme heterogeneity in true effect size [5, 32, 50, 52]. This implied that the population that we study is the homogeneous subsets of meta-analyses that were published in the psychological and medical literature. Drawbacks of examining heterogeneity in true effect size with the *I*^2^-statistic are that its value heavily depends on the sample size of the primary studies in case of heterogeneity [86] and the statistic is imprecise in case of a small number of primary studies in a meta-analysis [87, 88]. However, the *I*^2^-statistic enables comparison across meta-analyses that used different effect size measures which is not possible by comparing estimates of the between-study variance (*τ*^2^) in true effect size of meta-analyses. Different effect size measures were sometimes used within a meta-analysis. This may cause heterogeneity in a meta-analysis, so the type of effect size measure was also used for creating homogeneous subsets. Publication bias tests have low statistical power (e.g., [5, 47, 89]) if the number of effect sizes in a meta-analysis is small. Hence, another criterion for including a subset in the analyses was that a subset should contain at least five effect sizes.

**Fig 2:**
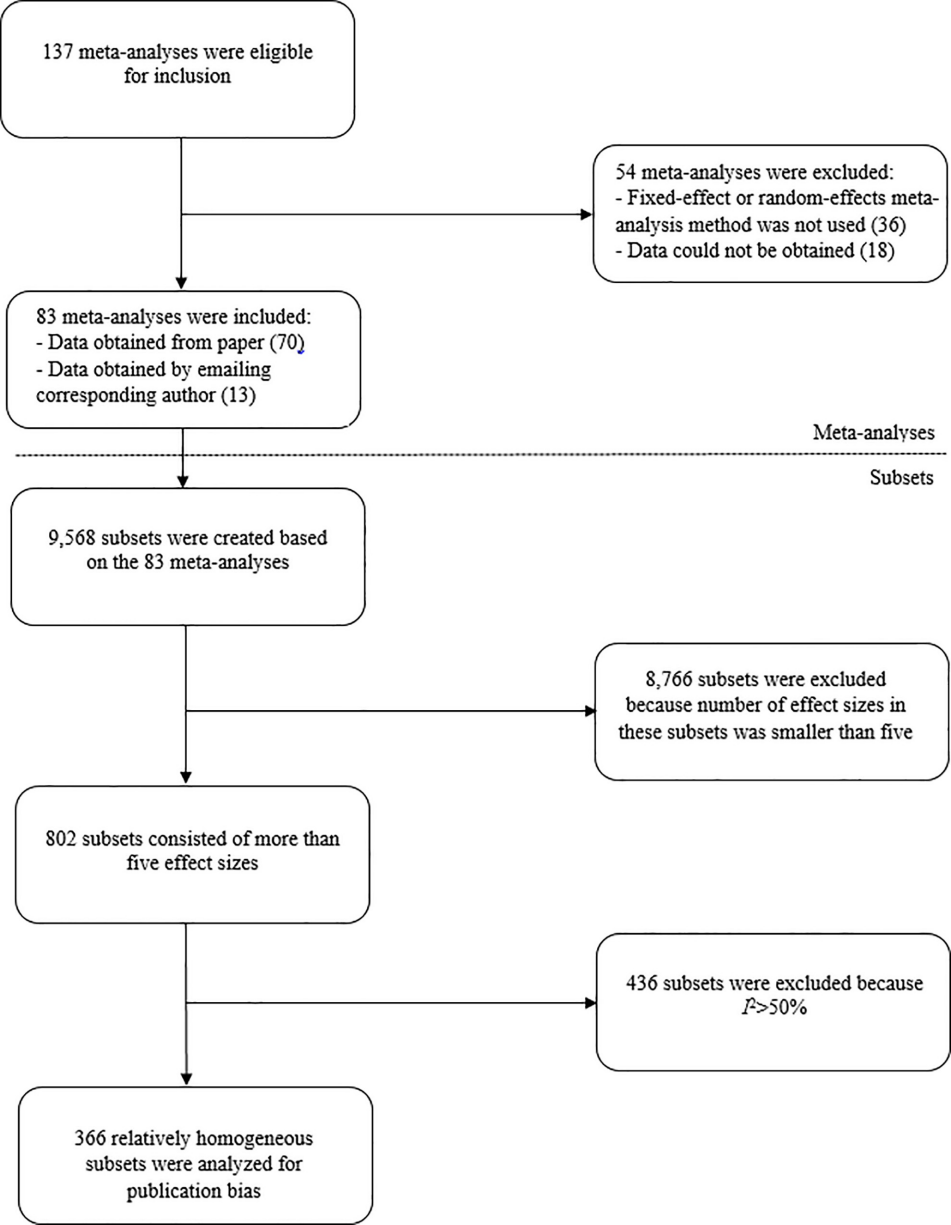
Flowchart illustrating the extraction procedure of data from meta-analyses published in Psychological Bulletin between 2004 and 2014.

We searched within the journal Psychological Bulletin for meta-analyses published between 2004 and 2014 by using the search terms “meta-analy*” and *not* “comment”, “note”, “correction”, and “reply” in the article’s title. This search resulted in 137 meta-analyses that were published between 2004 and 2014 and that were eligible for inclusion. A flowchart is presented in Fig 2 describing the data extraction for the meta-analyses published in Psychological Bulletin. Eighty-three meta-analyses met the inclusion criteria and could be included since the data were available in the paper or were obtained by emailing the corresponding author. Data of these meta-analyses were extracted by hand and resulted in 9,568 subsets. Data from a random sample of 10% of the included meta-analyses was extracted a second time by a different researcher to verify the procedure of extracting data. Four additional subsets were excluded after verifying the data, because these subsets were heterogeneous instead of homogeneous. After excluding subsets with less than five effect sizes and heterogeneous subsets, a total number of 366 subsets from 83 meta-analyses were available for the analyses.

Data of all systematic reviews in the CDSR are stored online in a standardized format, and data of these reviews can therefore be extracted by an automated procedure. We used the Cochrane scraper developed by Springate and Kontopantelis [90] to automatically extract data from systematic reviews. The total number of meta-analyses in the CDSR is larger than in Psychological Bulletin, so we drew a simple random sample without replacement of systematic reviews from the CDSR to represent meta-analyses published in medicine. Each systematic review in the database has an identification number. We sampled identification numbers, extracted subsets from the sampled systematic review, and included a subset in our study if (i) *I*^2^<50%, (ii) the number of effect sizes in a subset was at least five, and (iii) the subset was independent of previous included subsets (i.e., no overlap between effect sizes in different subsets). We continued sampling systematic reviews and extracting subsets till the same number of eligible subsets for inclusion were obtained as extracted from Psychological Bulletin (366). Data and/or descriptions of the data of the meta-analyses are available at https://osf.io/9jqht/. The next section describes how the research questions were answered, and how the variables were measured.

### Analysis

#### Prevalence of publication bias

The prevalence of publication bias in homogeneous subsets from meta-analyses in the psychological and medical literature was examined to answer research question 1 by using the methods listed in the last column of Table 1. Egger’s test and the rank-correlation test were used in the analyses to test for funnel plot asymmetry instead of eyeballing a funnel plot. *P-*uniform’s publication bias test can be applied to observed effect sizes in a subset that are either significantly smaller or larger than zero. Hence, *p*-uniform was applied to negative or positive statistically significant effect sizes in a subset depending on where the majority of statistically significant effect sizes was observed (using a two-tailed hypothesis test with α = .05). The estimator based on the Irwin-Hall distribution was used for *p*-uniform, because this estimator seemed to have the best statistical properties and provides a confidence interval [52]. Publication bias tests have low statistical power, so we followed a recommendation by Egger and colleagues [43] to conduct two-tailed hypothesis tests with α = .1 for all methods. Unintentionally, one-tailed *p*-values of *p*-uniform’s publication bias test were computed in the preregistered R code for subsets of CDSR instead of the intended two-tailed *p-*values. Since two-tailed *p-*values were computed for all the other publication bias tests, we corrected the pre-registered R code such that two-tailed *p-*values were also computed for *p*-uniform’s publication bias test.

We answered research question 1a about the prevalence of publication bias in meta-analyses published in Psychological Bulletin and CDSR by counting how often each method rejects the null hypothesis of no publication bias. Agreement among the publication bias tests was examined by computing Loevinger’s *H* values [91] for each combination of two methods. Loevinger’s *H* is a statistic to quantify the association between two dichotomous variables (i.e., statistically significant or not). The maximum value of Loevinger *H* is 1 indicating a perfect association where the minimum value depends on characteristics of the data. For subsets with no statistically significant effect sizes, *p-*uniform could not be applied, so we computed the association between the results of *p-*uniform and other methods only for subsets with statistically significant effect sizes.

We studied whether publication bias was more prevalent in homogeneous subsets from Psychological Bulletin and CDSR (research question 1b) by conducting for each publication bias test a logistic regression with as dependent variable whether a publication bias test was statistically significant or not and as predictor a dummy variable indicating whether a subset was obtained from Psychological Bulletin or CDSR (reference category). The number of effect sizes in a subset (or statistically significant effect sizes for *p*-uniform) was included as control variable, because statistical power of publication bias tests depends on the number of effect sizes in a subset and the number of effect sizes in subsets from meta-analyses published in Psychological Bulletin and CDSR were expected to differ. We hypothesized that publication bias would be more severe in subsets from Psychological Bulletin than CDSR after controlling for the number of effect sizes in a subset (or number of statistically significant effect sizes for *p*-uniform). This relationship was expected because medical researchers have been longer aware of the consequences of publication bias whereas broad awareness of publication bias recently originated in psychology. One-tailed hypothesis tests with α = .05 were used for answering research question 1b. As a sensitivity analysis, we also conducted for each publication bias test a multilevel logistic regression where we take into account that the subsets were nested in the meta-analyses. This analysis was not specified in the pre-analysis plan.

#### Predicting effect size estimation

Characteristics of subsets were used to predict the estimates of random-effects meta-analysis and estimates of *p-*uniform in research question 2. All effect sizes and their sampling variances were transformed to Cohen’s *d* to enable interpretation of the results by using the formulas in section 12.5 of [82]. If Cohen’s *d* and their sampling variances could not be computed based on the available information, Hedges’ *g* was used as an approximation of Cohen’s *d* (6.4% of all subsets).

Random-effects meta-analysis was used to estimate the effect size rather than fixed-effect meta-analysis. Random-effects meta-analysis assumes that there is no single fixed true effect underlying each effect size [92], and was preferred over fixed-effect meta-analysis because a small amount of heterogeneity in true effect size could be present in the subsets. The Paule-Mandel estimator [93] was used in random-effects meta-analysis for estimating the amount of between-study variance in true effect size since this estimator has the best statistical properties in most situations [94, 95]. Effect sizes corrected for publication bias were estimated with *p*-uniform and based on the 10% most precise observed effect sizes (see last column of Table 1). Estimation based on the 10% most precise observed effect sizes was included as an exploratory analysis to examine whether estimates of *p-*uniform were in line with another method to correct effect sizes for publication bias. If the number of observed effect sizes in a subset was smaller than ten, the most precise estimate was interpreted as estimate of the 10% most precise observed effect sizes. For applying *p*-uniform, the estimator based on the Irwin-Hall distribution was used, and two-tailed hypothesis tests in the primary studies were conducted with α = .05. The underlying true effect size in a subset can be either positive or negative. Hence, the dependent variables of these analyses were the absolute values of the estimates of random-effects meta-analysis and *p*-uniform.

Selection model approaches and PET-PEESE methods were not incorporated in the analyses, because the number of effect sizes included in meta-analyses in medicine is often too small for these methods. Selection model approaches suffer from convergence problems when applied to data with these characteristics (e.g., [60, 61]), and PET-PEESE is not recommended to be used since it yields unreliable results if there are less than 10 observed effect sizes [56]. *P-*uniform was preferred over trim and fill and *p-*curve, because applying trim and fill is discouraged [5, 53, 54] and *p*-curve is not able to estimate a confidence interval around its effect size estimate.

Two weighted least squares (WLS) regressions were performed with as dependent variables the absolute values of the effect size estimates of either random-effects meta-analysis or *p*-uniform. Since we meta-analyze the effect sizes estimated with meta-analysis methods, we refer to these analyses as meta-meta-regressions. The inverse of the variance of a random-effects model was selected as weights in both meta-meta-regressions, because it is a function of both the sample size of the primary studies and the number of effect sizes in a subset. *P-*uniform can only be applied to subsets with statistically significant effect sizes, so the meta-meta-regression with the effect size estimates of *p*-uniform as dependent variable was only based on these subsets.

Four predictors were included in the meta-meta regressions. The predictors and the hypothesized relationships are listed in the first two columns of Table 2. The meta-meta-analytic effect size estimate was expected to be larger in subsets from Psychological Bulletin, because publication bias was expected to be more severe in psychology than medicine. No relationship was hypothesized between the *I*^2^-statistic and the meta-analytic effect size estimate, because heterogeneity can be either over- or underestimated depending on the extent of publication bias [96, 97]. Primary studies’ precision in a subset was operationalized by computing the harmonic mean of the primary studies’ standard error. A negative relationship was expected between primary studies’ precision and the meta-analytic estimate, because less precise effect size estimates (i.e., larger standard errors) were expected to be accompanied with more bias and hence larger meta-analytic effect size estimates. The number of effect sizes in a subset was included to control for differences in the number of studies in a meta-analysis.

**Table 2:** Hypotheses between predictors and effect size estimate based on random-effects model, *p-*uniform, and overestimation in effect size when comparing estimate of the random-effects model with *p*-uniform (*Y*).

	Hypotheses		
Predictor	Random-effects model	*p-*uniform	Overestimation (*Y*)
Discipline	Larger estimates in subsets from Psychological Bulletin	No specific expectation	Overestimation more severe in Psychological Bulletin
*I*^2^-statistic	No relationship	Positive relationship	Negative relationship
Primary studies’ precision	Negative relationship	No relationship	Negative relationship
Proportion of significant effect sizes	Predictor not included	No specific expectation	No specific expectation

The hypotheses concerning the effects in the meta-meta regression on *p*-uniform’s estimates are presented in the third column of Table 2. No hypothesis was specified for the effect of discipline since *p*-uniform is supposed to correct for possible differences between both disciplines in effect sizes due to publication bias. We expected a positive relationship with the *I*^2^-statistic, because *p*-uniform overestimates the true effect size in the presence of heterogeneity in true effect size [5, 52]. No specific relationship was predicted with primary studies’ precision as *p*-uniform is supposed to correct for publication bias. A specific relationship was also not hypothesized for the effect of the proportion of statistically significant effect sizes in a subset. Many statistically significant effect sizes in a subset suggest that the studied effect size is large, sample size of the primary studies are large, or there was severe publication bias in combination with many conducted (but not published) primary studies. These partly opposing effects might have canceled each other out or there can be a positive or negative relationship. The number of effect sizes in a subset was again included as control variable.

The effect size estimate of *p*-uniform can become extremely positive or negative if there are multiple *p*-values just below the α-level [5, 52]. These outliers may affect the results of the meta-meta-regression with *p*-uniform’s estimate as dependent variable. Hence, we used quantile regression [98] as a sensitivity analysis, because this procedure is less influenced by outliers in the dependent variable. In quantile regression, the predictors were regressed on the median of the estimates of *p*-uniform. Moreover, we also conducted another meta-meta-regression as a sensitivity analysis where we added a random effect to take into account that the subsets were nested in meta-analyses. Both sensitivity analyses were exploratory analyses that were not specified in the pre-analysis plan.

#### Overestimation of effect size

Estimates of random-effects meta-analysis and *p*-uniform obtained for answering research question 2 were used to examine the overestimation caused by publication bias. As an exploratory analysis, overestimation was also studied by comparing estimates of random-effects meta-analysis with those of 10% most precise observed effect sizes. It is possible that especially estimates of the meta-analysis and *p*-uniform have opposite signs (i.e., negative estimate of *p*-uniform and positive meta-analytic estimate or the other way around). An effect size estimate of *p-*uniform in the opposite direction than the meta-analytic estimate is often unrealistic, because this suggests that, for instance, a negative true effect size results in multiple positive observed effect sizes. Effect size estimates in opposing directions by meta-analysis and *p-*uniform may be caused by many *p-*values just below the α-level [52]. Hence, *p*-uniform’s estimate was set equal to zero in these situations. Setting *p-*uniform’s estimate to zero when its sign is opposite to that of random-effects meta-analysis is in line with the recommendation in [52]. We did not set estimates based on the 10% most precise observed effect sizes to zero, because this estimator will not yield unrealistic estimates in the opposite direction than random-effects meta-analysis in the absence of heterogeneity. Such an estimate in the opposite direction based on the 10% most precise observed effect sizes is also unlikely to occur. The most precise observed effect sizes get the largest weight in a random-effects meta-analysis and the sign of these precise observed effect sizes is for the vast majority of cases in line with the sign of the random-effects meta-analysis.

A new variable *Y* was created to reflect the overestimation of random-effects meta-analysis when compared with *p*-uniform and the 10% most precise observed effect sizes. Such a *Y*-variable was created for both methods that correct effect size estimates for publication bias. If the meta-analytic estimate was larger than zero, *Y* = MA-corrected where “MA” is the meta-analytic estimate and “corrected” is the estimate of either *p*-uniform or the 10% most precise observed effect sizes. If the meta-analytic estimate was smaller than zero, *Y* = -MA+corrected. Variable *Y* was zero if the estimates of the random-effects meta-analysis and an estimate corrected for publication bias were the same, positive if a corrected effect size estimate was closer to zero than the meta-analytic estimate (if they originally had the same sign), and negative if a corrected estimate was farther away from zero than the meta-analytic estimate (if they originally had the same sign). The *Y* variable based on *p-*uniform was computed for each subset with statistically significant effect sizes. We computed the mean, median, and a 95% confidence interval by using a normal approximation and estimated standard error equal to the standard deviation of *Y* divided by the square root of the number of homogeneous subsets. These estimates and 95% confidence intervals were computed for subsets from Psychological Bulletin and CDSR in order to gather insight in the amount of overestimation in effect size (research question 3a).

To answer research question 3b, we carried out meta-meta regressions on *Y* based on *p-u*niform with the inverse of the variance of the random-effects meta-analytic estimate as weights. We used the predictors that we also included in research question 2. The hypothesized relationships are summarized in the fourth column of Table 2. A larger value on *Y* was expected for subsets from Psychological Bulletin than CDSR, because overestimation was expected to be more severe in psychology than in medicine. We hypothesized a negative relation between the *I*^2^-statistic and *Y*, because *p-*uniform overestimates the effect size in the presence of heterogeneity in true effect size [5, 52]. Primary studies’ precision was hypothesized to be negatively related to *Y*, because overestimation of the meta-analytic estimate was expected to decrease as a function of primary studies’ precision. We had no specific expectations on the relationships between the number of effect sizes in a subset and the proportion of statistically significant effect sizes in a subset. Although a positive effect of this proportion on the meta-analytic effect size estimate was expected, the effect of the proportion on *p*-uniform’s estimate was unclear. We included the number of effect sizes in a subset in the meta-meta-regression as a control variable.

Estimates of *p-*uniform that were in the opposite direction than traditional meta-analysis were set equal to zero before computing the *Y*-variable. This may have affected the results of the meta-meta-regression since the dependent variable *Y* did not follow a normal distribution. Hence, quantile regression [98] was used as sensitivity analysis with the median of *Y* as dependent variable instead of the mean of *Y* in the meta-meta regression. We also conducted another meta-meta-regression as a sensitivity analysis where a random effect was included to take into account that the subsets were nested in meta-analyses. Both sensitivity analyses were exploratory analyses that were not specified in the pre-analysis plan.

#### Monte-Carlo simulation study

Following up on the comments of a reviewer we examined the statistical properties of our preregistered analyses by means of a Monte-Carlo simulation study. More specifically, we examined the statistical power of publication bias tests and properties of effect size estimation based on the 10% most precise observed effect sizes, both as a function of publication bias and true effect size. As the analysis based on the 10% most precise estimates does not make any assumptions about the publication process (like the publication bias methods, including *p*-uniform), we consider this analysis to provide additional valuable information about the extent of publication bias in the psychology and medicine literature.

Cohen’s *d* effect sizes were simulated under the fixed-effect meta-analysis model using the number of observed effect sizes and their standard errors of the homogeneous subsets included in our large-scale dataset. That is, effect sizes were simulated from a normal distribution with mean μ and variance equal to the ‘observed’ squared standard errors of each homogeneous subset. Publication bias was introduced by always including statistically significant effect sizes where significance was determined based on a one-tailed test with α = .025 to resemble common practice to test a two-tailed hypothesis with α = .05 and only report results in the predicted direction. All generated nonsignificant effect sizes had a probability equal to 1-*pub* to be included. For each effect size in the homogeneous subset, the observed effect size was simulated until it was ‘published’; as a result the simulated homogeneous subset had the same properties (number of studies, standard errors of the studies but not the effect sizes and their corresponding *p-*values) as the observed homogeneous subset.

The publication bias tests (see Table 1 for the included methods) and methods to correct effect size for publication bias (*p*-uniform and meta-analysis based on the 10% most precise observed effect sizes) were applied to data of each generated homogeneous subset. We examined Type-I error rate and statistical power of the publication bias tests using the same α-level (i.e., 0.1) as for testing for publication bias in the homogeneous subsets. We also assessed the overestimation of the random-effects model with the Paule-Mandel estimator [93] for the between-study variance when compared with the 10% most precise observed effect sizes by computing the earlier introduced *Y*-variable.

Data of homogeneous subsets were simulated for characteristics of all 732 homogeneous subsets and repeated 1,000 times. Values for μ were selected to reflect no (μ = 0), small (μ = 0.2), and medium (μ = 0.5) effect regarding the guidelines by Cohen [99]. Publication bias (*pub*) was varied from 0, 0.25, 0.5, 0.75, 0.85, 0.95, and 1, with *pub* = 0 implying no publication bias and 1 extreme publication bias. The Monte-Carlo simulation study was programmed in R [100] and the packages “metafor” [101], “puniform” [102], and “parallel” [100] were used (see https://osf.io/efkn9/ for R code of the simulation study).

## Results

### Descriptive statistics

The total number of included homogeneous subsets was 732 (366 representing Psychological Bulletin and 366 representing CDSR). Table 3 shows descriptive results (number of effect sizes, percentage of statistically significant effect sizes, primary study sample sizes, and positive and negative meta-analytic effect size estimates) of applying random-effects meta-analysis, *p*-uniform, and random-effects meta-analysis based on the 10% most precise observed effect sizes.

**Table 3:** Percentage of statistically significant effect size estimates, median number of effect sizes and median of average sample size per homogeneous subset, and mean and median of effect size estimates when the subsets were analyzed with random-effects meta-analysis, *p*-uniform, and random-effects meta-analysis based on the 10% most precise observed effect sizes.

			RE meta-analysis	*p*-uniform	10% most precise
**Psychological Bulletin** **28.9% statistically significant**					
		Median (IQR) number of effect sizes	6 (5;9)	1 (0;4)	1 (1;1)
		Median (IQR) sample size	97.8 (52.4;173.2)	109 (56.5;206.2)	207.3 (100;466)
	Positive RE meta-analysis estimates: 67.2% of homogeneous subsets				
		Mean, median, [min.;max.], (SD) of estimates	0.332, 0.279, [0;1.456] (0.264)	-0.168, 0.372, [-21.584;1.295] (2.367)	0.283, 0.22, [-0.629;1.34] (0.289)
	Negative RE meta-analysis estimates: 32% of homogeneous subsets				
		Mean, median, [min.;max.], (SD) of estimates	-0.216, -0.123, [-1.057;-0.002] (0.231)	-0.041, -0.214, [-5.166;13.845] (1.84)	-0.228, -0.204, [-0.972;0.181] (0.247)
**CDSR** **18.9% statistically significant**					
		Median (IQR) number of effect sizes	6 (5;8)	1 (0;2)	1 (1;1)
		Median (IQR) sample size	126.6 (68.3;223.3)	123.3 (71.9;283.5)	207 (101.2;443)
	Positive RE meta-analysis estimates: 45.1% of homogeneous subsets				
		Mean, median, [min.;max.], (SD) of estimates	0.304, 0.215, [0.001;1.833] (0.311)	-1.049, 0.323, [-60.85;1.771] (6.978)	0.284, 0.201, [-0.709;1.757] (0.366)
	Negative RE meta-analysis estimates: 54.9% of homogeneous subsets				
		Mean, median, [min.;max.], (SD) of estimates	-0.267, -0.19, [-1.343;0] (0.253)	1.51, -0.239, [-1.581;163.53] (15.064)	-0.214, -0.182, [-1.205;0.644] (0.286)

RE meta-analysis is random-effects meta-analysis, IQR is the interquartile range, min. is the minimum value, max. is the maximum value, SD is the standard deviation, and CDSR is Cochrane Database of Systematic Reviews. The percentages of homogeneous subsets with positive and negative RE meta-analysis estimates do not sum to 100%, because the estimates of three homogeneous subsets obtained from the meta-analysis by Else-Quest and colleagues [103] were equal to zero. These authors set effect sizes to zero if the effect size could not have been extracted from a primary study but was reported as not statistically significant.

The percentage of effect sizes (across all homogeneous subsets) that was statistically significant was 28.9% and 18.9% in Psychological Bulletin and CDSR, respectively. These percentages were lower than those based on the excluded heterogeneous subsets (44.2% and 28.9%, respectively). The number of effect sizes in subsets was similar in Psychological Bulletin and CDSR. The majority of subsets contained less than 10 effect sizes (third quartile 9 for Psychological Bulletin and 8 for CDSR) meaning that the characteristics of the subsets were very tough for publication bias methods. Statistical power of publication bias is low in these conditions [47, 49] and effect size estimates corrected for publication bias are imprecise [5, 52]. The number of statistically significant effect sizes in the subsets based on a two-tailed hypothesis test with α = .05 was also small (listed in column with results of *p*-uniform). The median number of statistically significant effect sizes in the subsets was 1 for both Psychological Bulletin and CDSR. Moreover, 267 (73%) of the subsets from Psychological Bulletin and 214 (58.5%) of the subsets from CDSR contained at least one statistically significant effect size; hence 27% and 41.5% of subsets did not contain a single statistically significant effect size. Consequently, *p*-uniform could only be applied to 481 (65.7%) of the subsets. Of these subsets 180 (37.4%) included only one statistically significant effect size, so the characteristics of the subsets were very challenging for *p*-uniform. However, methods based on similar methodology as *p-*uniform to, for instance, compare an original study and replication and to determine the required sample size in a power analysis showed that one or two effect sizes can be sufficient for accurate estimation of effect size [5, 104–106]. The median and interquartile range of the 10% most precise effect size estimates were all equal to one, and estimates of this method were for 676 (92.3%) subsets based on only one effect size.

The median of the average sample size per subset was slightly larger for CDSR (126.6) than for Psychological Bulletin (97.8). The interquartile range of average sample size within subsets from CDSR (68.3; 223.3) was also larger than for subsets from Psychological Bulletin (52.4;173.2). Psychological Bulletin and CDSR showed small differences in the median and interquartile range of the average sample size in subsets if computed based on only the statistically significant effect sizes (*p-*uniform) or the 10% most precise effect size estimates.

Results of estimating effect size in subsets with random-effects meta-analysis, *p*-uniform, and random-effects meta-analysis based on the 10% most precise observed effect sizes (exploratory analysis) are also included in Table 3. To increase interpretability of the results, estimates were grouped depending on whether the effect size estimate of random-effects meta-analysis was positive or negative. The mean and median of the effect size estimates of random-effects meta-analysis and those based on the 10% most precise observed effect sizes were highly similar (difference at most 0.053). However, estimates of *p*-uniform deviated from the other two methods, because *p*-uniform’s estimates were in some subsets very positive or negative (i.e., 4 estimates were larger than 10 and 7 estimates were smaller than -10) due to *p*-values of the primary study’s effect sizes close to the α-level. Consequently, the standard deviation and range of the estimates of *p*-uniform were larger than of random-effects meta-analysis and based on the 10% most precise observed effect sizes.

### Prevalence of publication bias

Table 4 shows the results of applying Egger’s regression test, the rank-correlation test, *p*-uniform’s publication bias test, and the TES to examine the prevalence of publication bias in the meta-analyses. The panels in Table 4 illustrate how often each publication bias test was statistically significant (marginal frequencies and percentages) and also the agreement among the methods (joint frequencies). Agreement among the methods was quantified by means of Loevinger’s *H* (bottom-right cell of each panel).

**Table 4:** Results of applying Egger’s regression test, rank-correlation test, *p*-uniform’s publication bias test, and test of excess significance (TES) to examine the prevalence of publication bias in meta-analyses from Psychological Bulletin and Cochrane Database of Systematic Reviews.

		Rank-correlation					*p*-uniform		
		Not sig.	Sig.				Not sig.	Sig.	
Egger	Not sig.	600	35	635; 87.1%	Egger	Not sig.	354	34	388; 83.3%
	Sig.	51	43	94; 12.9%		Sig.	70	8	78; 16.7%
	Total	651; 89.3%	78; 10.7%	*H* = .485		Total	424; 91%	42; 9%	*H* = .028
		TES					*p*-uniform		
		Not sig.	Sig.				Not sig.	Sig.	
Egger	Not sig.	609	29	638; 87.2%	Rank-corr.	Not sig.	377	34	411; 88.2%
	Sig.	83	11	94; 12.8%		Sig.	47	8	55; 11.8%
	Total	692; 94.5%	40; 5.5%	*H* = .168		Total	424; 91%	42; 9%	*H* = .082
		TES					TES		
		Not sig.	Sig.				Not sig.	Sig.	
Rank-corr.	Not sig.	620	31	651; 89.3%	*p*-uniform	Not sig.	393	31	424; 91%
	Sig.	69	9	78; 10.7%		Sig.	33	9	42; 9%
	Total	689; 94.5%	40; 5.5%	*H* = .132		Total	426; 91.4%	40; 8.6%	*H* = .148

*H* denotes Loevinger’s *H* to describe the association between two methods. The rank-correlation could not be applied to all 732 subsets, because there was no variation in the observed effect sizes in three subsets. All these subsets were part of the meta-analysis by Else-Quest and colleagues [103] who set effect sizes to zero if the effect size could not have been extracted from a primary study but was reported as not statistically significant.

Publication bias was detected in at most 94 subsets (12.9%) by Egger’s regression test. The TES and rank-correlation test were statistically significant in 40 (5.5%) and 78 (10.7%) subsets, respectively. In the subsets with at least one statistically significant effect size, *p*-uniform’s publication bias test detected publication bias in 42 subsets (9%), which was more than TES (40; 8.6%) and less than both the rank-correlation test (55; 11.8%) and Egger’s regression test (78; 16.7%). Since the estimated prevalence values are close to 10%, which equals the significance threshold of each test, we conclude there is at best weak evidence of publication bias on the basis of publication bias tests. Associations among the methods were low (*H <* .168), except for the association between Egger’s regression test and the rank-correlation test (*H* = .485).

To answer research question 1b we examined whether publication bias was more prevalent in subsets from Psychological Bulletin than CDSR. Publication bias was detected in 13.4% (Egger’s test), 12.8% (rank-correlation test), 11.4% (*p-*uniform), 6.6% (TES) of the subsets from Psychological Bulletin and in 12.2% (Egger’s test), 8.5% (rank-correlation test), 5.9% (*p*-uniform), and 4.4% (TES) of the subsets from CDSR. When testing for differences in publication bias we controlled for the number of effect sizes (or for *p-*uniform statistically significant effect sizes) in a meta-analysis. Publication bias was more prevalent in subsets from Psychological Bulletin if the results of *p*-uniform were used as dependent variable (odds ratio = 2.226, *z* = 2.217, one-tailed *p*-value *=* .014), but not for Egger’s regression test (odds ratio = 1.024, *z* = 0.106, one-tailed *p*-value *=* .458), rank-correlation test (odds ratio = 1.491, *z =* 1.613, one-tailed *p*-value *=* .054), and TES (odds ratio = 1.344, *z* = 0.871, one-tailed *p*-value *=* .192). Tables with the results of these logistic regression analyses are reported in S1–S4 Tables. Note, however, that if we control for the number of tests performed (i.e., 4) by means of the Bonferoni correction (*p* = .005 < .05/4 = .0125), the result of *p*-uniform was no longer statistically significant.

We also conducted multilevel logistic regression analyses to take into account that the subsets were nested in meta-analyses. The intraclass correlation can be used to assess to what extent the subsets within a meta-analysis were related to each other. These intraclass correlations were 14.9%, 25.6%, 0%, and 0% for Egger’s test, the rank-correlation test, *p-*uniform, and TES, respectively. Taking into account the nested structure hardly affected the parameter estimates and did not change the statistical inference (see S5–S8 Tables). All in all, we conclude that evidence of publication bias was weak at best and that we found no evidence of a difference in the extent of publication bias existed between subsets from Psychological Bulletin and CDSR.

### Predicting effect size estimation

To answer research question 2, absolute values of the effect size estimates of random-effects meta-analysis and *p*-uniform were predicted based on characteristics of the subsets. One-tailed hypothesis tests were used in case of a directional hypothesis (see Table 2 for a summary of our hypotheses). Table 5 presents the results of the meta-meta-regression on the absolute value of the effect size estimates of random-effect meta-analysis. The variables in the model explained 15.2% of the variance in the estimates of random-effects meta-analysis (*R*^2^ = 0.152; *F*(4,727) = 32.6, *p* < .001). The absolute value of the meta-analytic estimate was 0.056 larger for subsets from Psychological Bulletin compared to CDSR, and this effect was statistically significant and in line with our hypothesis (*t*(727) = 3.888, *p* < .001, one-tailed). The *I*^2^-statistic had an unexpected positive association with the absolute value of the meta-analytic estimate (B = 0.002, *t*(727) = 3.927, *p* < .001, two-tailed). The harmonic mean of the standard error had, as expected, a positive effect (B = 0.776, *t*(727) = 10.685, *p* < .001, one-tailed). The intraclass coefficient that was obtained with the sensitivity analysis where a random effect was included to take into account that the subsets were nested in meta-analyses was equal to 1.1%. The results of this sensitivity analysis are shown in S9 Table and were highly similar to the results of the analyses where the hierarchical structure was not taken into account.

**Table 5:** Results of meta-meta regression on the absolute value of the random-effects meta-analysis effect size estimate with predictors discipline, *I*^2^-statistic, harmonic mean of the standard error (standard error), and number of effect sizes in a subset.

	B (SE)	*t-*value (*p*-value)	95% CI
Intercept	0.035 (0.018)	1.924 (.055)	-0.001;0.07
Discipline	0.056 (0.014)	3.888 (< .001)	0.028;0.084
*I*^2^-statistic	0.002 (0.0004)	3.927 (< .001)	0.001;0.002
Standard error	0.776 (0.073)	10.685 (< .001)	0.633;0.918
Number of effect sizes	-0.002 (0.0005)	-4.910 (< .001)	-0.003;-0.001

CDSR is the reference category for discipline. *p-*values for discipline and harmonic mean of the standard error are one-tailed whereas the other *p-*values are two-tailed. CI = Wald-based confidence interval.

Table 6 shows the results of meta-meta regressions on the absolute value of *p-*uniform’s estimate as the dependent variable. The proportion explained variance in *p*-uniform’s estimate was *R*^2^ = .014 (*F*(5,475) = 1.377, *p* = .231). None of the predictors was statistically significant. The results of the sensitivity analysis where a random effect was included to take into account that the subsets were nested in meta-analyses were highly similar (see S10 Table). This was no surprise as the intraclass correlation was estimated as 0%. Quantile regression was used as sensitivity analysis to examine whether the results were distorted by extreme effect size estimates of *p*-uniform (see S11 Table). The results of the predictors discipline and *I*^*2*^-statistic were also not statistically significant in the quantile regression. The association of the harmonic mean of the standard error was lower in the quantile regression but statistically significant (B = 2.021, *t*(475) = 7.969, *p* < .001, two-tailed) and the predictor “proportion of statistically significant effect sizes” was statistically significant (B = 0.196, *t*(475) = 2.353, *p* = .019, two-tailed).

**Table 6:** Results of meta-meta-regression on the absolute value of *p-*uniform’s effect size estimate with predictors discipline, *I*^2^-statistic, harmonic mean of the standard error (standard error), proportion of statistically significant effect sizes in a subset (Prop. sig. effect sizes), and number of effect sizes in a subset.

	B (SE)	*t-*value (*p*-value)	95% CI
Intercept	0.77 (0.689)	1.118 (0.264)	-0.584;2.124
Discipline	0.001 (0.497)	0.001 (0.999)	-0.975;0.976
*I*^2^-statistic	0.013 (0.014)	0.939 (0.174)	-0.014;0.039
Standard error	3.767 (2.587)	1.456 (0.146)	-1.316;8.851
Prop. sig. effect sizes	-1.287 (0.797)	-1.615 (0.107)	-2.853;0.279
Number of effect sizes	-0.02 (0.015)	-1.363 (0.173)	-0.049;0.009

CDSR is the reference category for discipline. *p-*value for the *I*^2^-statistic is one-tailed whereas the other *p-*values are two-tailed. CI = Wald-based confidence interval.

### Overestimation of effect size

Table 7 shows descriptive statistics and 95% confidence intervals of the *Y*-variables comparing estimates of random-effects meta-analysis with *p-*uniform (first two columns) and the 10% most precise observed effect sizes (last two columns). Results of *p*-uniform suggest that possible overestimation because of publication bias was at most minimal for subsets from Psychological Bulletin: mean = -0.007, 95% CI = (-0.056;0.043), and median = 0.019. Overestimation for subsets from CDSR was larger but still small, and statistically significant (mean = 0.042, 95% CI = (0.002;0.083), median = 0.051). The overestimation based on the 10% most precise observed effect sizes was also small (*d*<0.04) and statistically significant for subsets from Psychological Bulletin (mean = 0.030, 95% CI = (0.011;0.048), median = 0.024) and CDSR (mean = 0.038, 95% CI = (0.016;0.061), median = 0.023). The slight overestimations provide indirect evidence of publication bias that appears to be similar in both fields (research question 3a).

**Table 7:** Mean, standard deviation (SD), 95% confidence interval (CI), and median of the *Y* variable computed with *p*-uniform and the 10% most precise observed effect sizes.

	*p-*uniform		10% most precise	
	Psy. Bull.	CDSR	Psy. Bull.	CDSR
Mean (SD)	-0.007 (0.412)	0.042 (0.305)	0.030 (0.181)	0.038 (0.220)
(95% CI)	(-0.056;0.043)	(0.002;0.083)	(0.011;0.048)	(0.016;0.061)
Median	0.019	0.051	0.024	0.023

Results are reported for homogeneous subsets of meta-analyses published in Psychological Bulletin (Psy. Bull.) and Cochrane Database of Systematic Reviews (CDSR).

Table 8 presents the results of the meta-meta regression on *Y* to answer research question 3b on predictors of the overestimation in effect size caused by publication bias. The predictors explained 11.8% of the variance of *Y* (*F*(5,475) = 12.76, *p* < .001). The effect size in subsets from Psychological Bulletin was not significantly larger than from CDSR (B = -0.040, *t*(475) = -1.651, *p* = .951, one-tailed). We found a negative effect of the *I*^2^-statistic on *Y* (B = -0.004, *t*(475) = -5.338, *p* < .001, one-tailed). The hypothesized relationship between the harmonic mean of the standard error and *Y* was not statistically significant (B = 0.172, *t*(475) = 1.371, *p =* .086, one-tailed). The proportion of statistically significant effect sizes in a subset was positively associated with *Y* (B = 0.182, *t*(475) = 4.713, *p* < .001, two-tailed).

**Table 8:** Results of meta-meta-regression on the effect size overestimation in random-effects meta-analysis when compared to *p-*uniform (*Y*) and predictors discipline, *I*^2^-statistic, harmonic mean of the standard error (standard error), proportion of statistically significant effect sizes in a subset (Prop. sig. effect sizes), and number of effect sizes in a subset.

	B (SE)	*t-*value (*p*-value)	95% CI
Intercept	-0.017 (0.033)	-0.517 (.605)	-0.083;0.048
Discipline	-0.04 (0.024)	-1.651 (.951)	-0.087;0.008
*I*^2^-statistic	-0.004 (0.001)	-5.338 (< .001)	-0.005;-0.002
Standard error	0.172 (0.126)	1.371 (.086)	-0.074;0.419
Prop. sig. effect sizes	0.182 (0.039)	4.713 (< .001)	0.106;0.258
Number of effect sizes	-0.001 (0.001)	-2.064 (.04)	-0.003;-0.0001

CDSR is the reference category for discipline. *p-*values for discipline, the *I*^2^-statistic, and the harmonic mean of the standard error are one-tailed whereas the other *p-*values are two-tailed. CI = Wald-based confidence interval.

The results of the sensitivity analysis where a random effect was included to take into account that subsets were nested in meta-analyses were highly similar (see S12 Table). Again, this was no surprise as the intraclass correlation coefficient was estimated as practically 0 (0.04%). Quantile regression with the median of *Y* as dependent variable was conducted to examine whether the results of the meta-meta-regression were affected by truncating the estimate of *p*-uniform to zero (see S13 Table). This truncation occurred in 136 subsets before computing *Y*. The predictor discipline was not statistically significant in the quantile regression. In contrast to the results of the meta-meta-regression, the effects of the *I*^2^-statistic (B = -0.0003, *t*(475) = -0.2, *p* = .579, one-tailed) and proportion of statistically significant effect size in a subset (B = -0.002, *t*(475) = -1.53, *p* = .127, two-tailed) were no longer statistically significant, whereas the predictor harmonic mean of the standard error was statistically significant (B = 0.279, *t*(475) = 1.889, *p* = .03, one-tailed).

### Monte-Carlo simulation study

**Fig 3:**
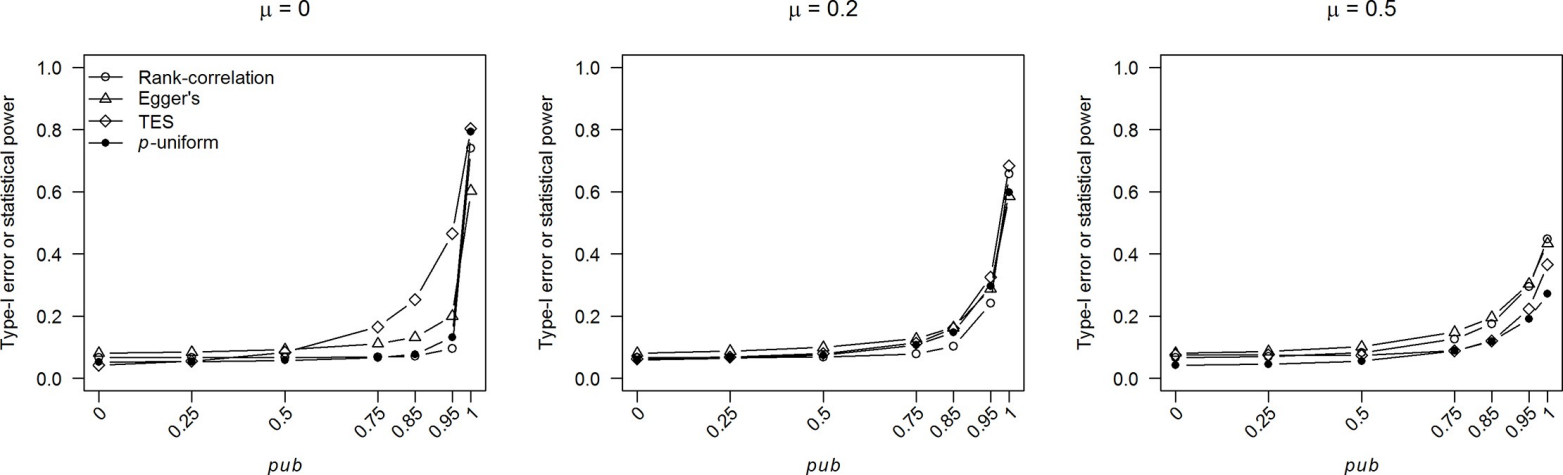
Type-I error rate and statistical power of the rank-correlation test (open bullets), Egger’s test (triangles), test of excess significance (TES; diamonds), and *p*-uniform’s publication bias test (solid bullets) in the Monte-Carlo simulation study. *pub* and μ are the extent of publication bias and the average true effect size, respectively.

Fig 3 shows the average Type-I error rate and the average statistical power across all 732 data sets of the rank-correlation test (open bullets), Egger’s test (triangles), the TES (diamonds), and *p*-uniform’s publication bias test (solid bullets) as a function of publication bias (*pub*) and average true effect size (μ). Average statistical power of all methods increased as a function of *pub* and decreased as a function of μ. None of the publication bias tests achieved an average statistical power larger than 0.5 if *pub* ≤ 0.95, and average statistical power did not exceed 0.2 for *pub* ≤ 0.75 for any true effect size examined.

Our Monte-Carlo simulation study confirms the low statistical power of the publication bias tests to detect publication bias in the small homogeneous subsets examined in this study. The results of the simulations also indicate that the observed proportions of statistically significant publication bias tests for the homogeneous subsets could have occurred for a large range of values for *pub*. The observed proportions of statistically significant results for each publication bias test (ranging from 0.055 to 0.167) are in line with publication bias varying from *pub* = 0 to *pub* = 0.95 for μ = 0 and 0.2 and *pub* = 0.85 for μ = 0.5. This means that, even in our large data set with 732 subsets, the proportion of statistically significant results of publication bias tests does not provide much information on the presence of publication bias in homogeneous subsets of meta-analyses as published in Psychological Bulletin and CDSR.

**Fig 4:**
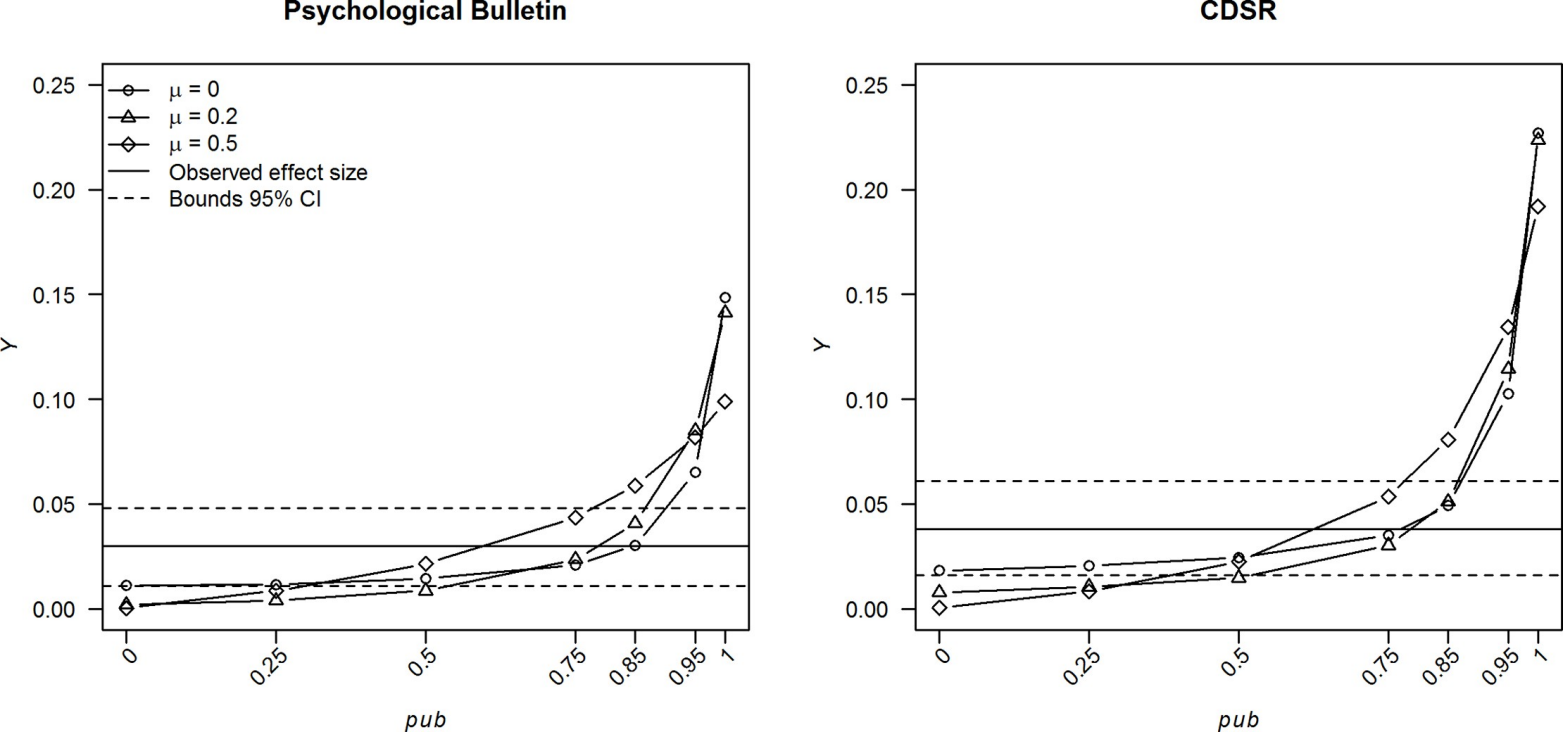
Overestimation (*Y*) of the random-effects model when compared with the 10% most precise observed effect sizes for simulated data based on characteristics of subsets from meta-analyses published in Psychological Bulletin (left panel) and Cochrane Database of Systematic Reviews (CDSR; right panel). *pub* refers to the extent of publication bias and open bullets, triangles, and diamonds indicate no, small, and medium average true effect size. The solid line indicates the mean of the *Y*-variable observed in the homogeneous subsets and the dashed lines are the upper and lower bound of a 95% confidence interval (CI) around the mean of the *Y*-variable.

Fig 4 illustrates the overestimation of effect size caused by publication bias for no (μ = 0; open bullets), small (μ = 0.2; triangles), and medium (μ = 0.5; diamonds) average true effects. We computed the mean of the *Y*-variable for each simulated homogeneous subset based on characteristics of subsets from meta-analyses published in Psychological Bulletin (left panel) and CDSR (right panel). The solid horizontal lines and dashed horizontal lines in Fig 4 refer to the mean and 95% confidence interval around this mean of the *Y*-variable observed in the homogeneous subsets (see Table 7). A larger value for *Y* refers to a larger overestimation by the random-effects model when compared with the 10% most precise observed effect sizes. Overestimation of effect size increased as a function of *pub*, but it was small for moderate publication bias (*pub* = 0.5) or even quite strong publication bias (*pub* < .85) for a zero true effect size.

The overestimation observed in the homogeneous subsets is in line with a large range of values for *pub*. For homogeneous subsets of meta-analyses published in Psychological Bulletin (left panel), the lower and upper bound of the 95% confidence intervals could be observed in combination with *pub* > 0.25 and < 0.95 for μ = 0, *pub* > 0.5 and < 0.95 for μ = 0.2, and *pub* > 0.25 and < 0.85 for μ = 0.5. The same holds for the subsets of meta-analyses from CDSR where the 95% confidence intervals could be observed when *pub* > 0 and < 0.95 for μ = 0, *pub* > 0.5 and < 0.95 for μ = 0.2, and *pub* > 0.25 and < 0.85 for μ = 0.5. Note, however, we should be careful with overinterpreting the results depicted in Fig 4 as estimated overestimation quite strongly depends on the assumed true effects, and we do not know the distribution of true effect size for the included homogeneous subsets. Nonetheless, we believe our Monte-Carlo simulation study revealed that the results in our study are in line with a wide range of publication bias, albeit not with scenarios where statistically nonsignificant effect sizes hardly ever get published. Specifically, our empirical results suggest at least mild publication bias as estimated overestimation was often not in the observed overestimation’s CI for *pub* ≤ 0.25 (Fig 4).

## Conclusion and discussion

Publication bias is a major threat to the validity of meta-analyses. It results in overestimated effect sizes in primary studies which in turn also biases the meta-analytic results (e.g., [3, 5]). Indications for the presence of publication bias have been observed in many research fields (e.g., [7, 8, 11, 14]), and different methods were developed to examine publication bias in a meta-analysis (for an overview see [2]). We studied the prevalence of publication bias and the overestimation caused by it in a large number of meta-analyses published in Psychological Bulletin and CDSR by applying publication bias methods to homogeneous subsets of these meta-analyses. Homogeneous subsets were created, because publication bias methods have poor statistical properties if the true effect size is heterogeneous [5, 32, 50, 52]. The prevalence of publication bias was studied by means of Egger’s test [43], the rank-correlation test [47], TES [50], and *p*-uniform’s publication bias test [5]. We used *p*-uniform and a meta-analysis based on the 10% most precise effect size estimates of a meta-analysis to estimate the effect size corrected for publication bias. The statistical properties of our preregistered analyses were also examined by means of a Monte-Carlo simulation study. Our paper is different from previous work [15–21] that studied the presence of questionable research practices and publication bias based on the distribution of *p-*values, because we did not analyze the distribution of *p*-values of studies published in a whole research field.

The results of our paper are not in line with previous research showing rather strong indications for publication bias in numerous research fields (e.g., [7, 8, 11, 14]) and revealing the presence of small-study effects in another meta-meta-analysis [107]. The best illustration of the diverging results of previous research and our study is that many (subsets of) meta-analyses did not contain a single statistically significant effect size (41.5% in CDSR and 27% in Psychological Bulletin), and only a minority of observed primary effect sizes was statistically significant (18.9% in CDSR and 28.9% in Psychological Bulletin). Although the percentage of statistically significant findings was slightly higher before excluding heterogeneous subsets (28.9% in CDSR and 44.2% in Psychological Bulletin), these percentages are substantially lower than the percentage of times the main hypothesis of a paper was deemed to be supported according to previous research. For example, approximately 90% of these hypotheses were statistically significant in the psychology and psychiatry [7, 8] and clinical medicine literature [108]. To put the percentages of statistically significant effect sizes in perspective, imagine that only 25% of all effects examined in a field have a nonzero true effect size and that statistical power equals 0.5 for a one-tailed hypothesis test with α = .025 to resemble researchers conducting a two-tailed test and reporting only the results in the predicted direction. Then 18.9% and 28.9% significant effects imply that 72% and 41.3% of nonsignificant effects get published, respectively (*pub* equals 0.28 and 0.587). Assuming higher prevalence of nonzero true effects, higher power, or assuming *p*-hacking implies *less* publication bias than calculated here. Thus, these numbers alone imply that if publication bias exists in homogeneous (subsets of) meta-analyses in psychology and medicine, then statistically nonsignificant effects still have a nonzero probability of getting published.

Only weak evidence for the prevalence of publication bias was observed in our large-scale data set of homogeneous subsets of primary studies. No evidence of bias was obtained using the publication bias tests. Overestimation was minimal but statistically significant (except for subsets from Psychological Bulletin in combination with *p*-uniform as estimator), providing evidence of publication bias that appeared to be similar in both fields. The simulation study (not preregistered) showed that the publication bias tests were only reasonably powered to detect extreme publication bias where all statistically nonsignificant effect sizes remain unpublished. No evidence for this extreme publication bias was present in our large data set. It also showed that the observed overestimation in the large data set was consistent with a wide range of relatively mild publication bias scenarios (but not with a scenario with extreme publication bias). Based on these findings in combination with the small percentages of statistically significant effect sizes in psychology and medicine, we conclude that evidence for publication bias in the studied homogeneous subsets is weak, but suggestive of mild publication bias in both disciplines.

A meta-meta-regression on the random-effects meta-analytic estimates revealed, in line with the hypothesis, a negative association of primary studies’ precision with a meta-analytic estimate. Since only weak evidence for publication bias was observed, this association was most likely mainly caused by differences in sample sizes between research fields. For instance, if researchers use statistical power analysis to determine the sample size of their study or if researchers in fields characterized by lower effect sizes use larger sample sizes by habit, larger true effect sizes will be associated with studies using smaller sample sizes (see supplemental materials of [44] and [45]).

The same predictors used for predicting the random-effects meta-analytic effect size estimate were also used in a meta-meta-regression on *p*-uniform’s estimate. None of the predictors statistically significantly predicted *p*-uniform’s effect size estimate. This was in line with our hypothesis on the relationship with primary studies’ precision, but in contrast to the expected positive relationship between the *I*^2^-statistic and *p*-uniform’s effect size estimate. The absence of such a positive relationship indicates that *p*-uniform did not overestimate the effect size in the presence of heterogeneity in true effect size. The initially unexpected positive association between the meta-analytic estimate and *I*^2^-statistic is in line with two very recent studies on multi-lab replication [109, 110], which showed absence of an effect in combination with homogeneous effect size, and heterogeneous effect sizes only in combination with some nonzero average effect size. The explained variance in the meta-meta-regression with *p*-uniform’s estimate as dependent variable (1.4%) was substantially lower than with the estimate of random-effects meta-analysis as dependent variable (67.6%). This difference in explained variance was mainly caused by the large variance in *p*-uniform’s effect size estimates across homogeneous subsets. The variance in these estimates was large, because estimates of *p*-uniform were based on a smaller subset of (statistically significant) effect sizes and were sometimes extremely positive or negative caused by observed effect sizes with *p*-values just below the α-level.

The different publication bias methods were not always in agreement with each other, which is caused by the absence of clear publication biases in the meta-analytic homogeneous subsets. An exception was the association between the results of Egger’s test and the rank-correlation test, but this association was expected since both methods are very similar in methodology (i.e., testing for publication bias by examining the relationship between observed effect size and some measure of its precision). Substantial differences were also observed among the two methods that we used to correct effect size estimates for publication bias (i.e., *p*-uniform and the 10% most precise observed effect size estimates). Effect size estimates based on the 10% most precise observed effect sizes were close to estimates of the random-effects meta-analysis whereas estimates of *p*-uniform were imprecise and sometimes very different from random-effects meta-analysis and the 10% most precise observed effect sizes. This suggests that *p-*uniform overcorrected for publication bias because of a small number of observed effect sizes in homogeneous subsets combined with *p*-values of primary studies being close to the α-level, because estimates based on the 10% most precise observed effect sizes are expected to be closer to *p*-uniform’s estimates in case of publication bias.

Our weak evidence of publication bias is at odds with meta-meta-analyses that found evidence for the presence of small-study effects (i.e., [107, 111]). One possible explanation for this disparity is that we created homogeneous subsets of primary studies in this paper. Small-study effects can be caused by heterogeneity in true effect size, so eliminating this heterogeneity by creating homogeneous subsets may have led to not observing small-study effects. Publication bias could, however, have gone undetected due to a variety of reasons. First, publication bias is less of an issue if the relationship of interest in a meta-analysis was not the main focus of the primary studies. Statistical significance of the main result in a primary study probably determines whether a result gets published, rather than whether a secondary outcome or supplementary result is significant. For instance, a meta-analysis might be about gender differences where data is extracted from studies that used gender only as a control variable. Second, meta-analysts included many unpublished studies in their meta-analyses, which might have decreased the severity and detectability of publication bias in our selected meta-analyses. Third, questionable research practices may have also decreased the detectability of publication bias in the meta-analyses, because questionable research practices may bias the effect size estimates of meta-analysis methods in any direction [52]. However, we do not believe that questionable research practices played a major role in the subsets in our paper, as relatively few effect sizes were statistically significant. The weak evidence for publication bias may be also caused by the challenging characteristics of the homogeneous subsets. Our Monte-Carlo simulation study revealed that publication bias tests did not achieve reasonable statistical power to detect moderate publication bias, and overestimation of effect size only became apparent in conditions where at most 1 out of 4 statistically nonsignificant effect size was included in a meta-analysis (*pub* ≥ 0.75).

Our focus on homogeneous (subsets of) meta-analyses limits the generalizability of our findings, because our conclusions can only straightforwardly be generalized to the population of subsets of primary studies of meta-analyses without evidence for medium or higher heterogeneity. However, limiting our population was necessary because publication bias methods’ statistical properties deteriorate if heterogeneity in true effect size is moderate or large. Determining whether primary studies in a subset were homogeneous or not was based on the *I*^2^-statistic. Homogeneous subsets that belong to the population under study can readily be identified in a meta-analysis, because the *I*^2^-statistic is now almost always reported in a meta-analysis [112] and routinely included in any Cochrane systematic review [87]. Another option for assessing the heterogeneity in true effect size is the *Q-*test for homogeneity [113], but this hypothesis test suffers from (too) low statistical power if a small number of primary studies are included in the meta-analysis [85, 114, 115]. However, drawbacks of the *I*^2^-statistic are that it heavily depends on the sample size of the primary studies [86] and the statistic is imprecise in case of a small number of primary studies in a meta-analysis [87, 88]. As a consequence, we may have wrongly included subsets that were heterogeneous and excluded subsets that were homogeneous. Moreover, our selection of homogeneous subsets could have led to the exclusion of subsets with severe publication bias. Imagine a subset with a number of statistically significant effect sizes that were published in a field with considerable publication bias, and a few statistically nonsignificant effect sizes that were obtained from unpublished research. The inclusion of the effect sizes from the unpublished research may cause heterogeneity in true effect size, and therefore a subset with potentially severe publication bias was excluded from our study.

Although no convincing evidence for publication bias was observed in our study, we agree with others (e.g., [34, 60, 116]) that publication bias should be routinely assessed in every meta-analysis. Moreover, a set of publication bias methods is recommended to be applied and reported in each meta-analysis, because each method assesses publication bias in a different way and one method might detect or correct for publication bias in a meta-analysis whereas another method might not [35, 36]. Future research should focus on developing publication bias methods that are able to examine publication bias in meta-analyses with heterogeneous true effect size, because effect size estimators that correct for publication bias are often biased in these conditions (e.g., [5, 52, 61, 62]). However, *p-*uniform was recently extended such that it can also deal with heterogeneous true effect size [106]. Other promising developments are the recently increased attention for selection model approaches (e.g., [61, 62, 106, 117]) and the development of a Bayesian method to correct for publication bias [80]. Albeit meta-analysts will greatly benefit from improved methods to assess publication bias, attention should also be paid to registering studies to make unpublished research readily accessible (e.g., [118]). Such a register enables meta-analysts to also include unpublished research in their meta-analysis and will improve the validity of meta-analytic results.

## Supporting information

S1 Table Results of logistic regression predicting statistical significance of Egger’s regression test with discipline and control variable number of effect sizes in a subset. (DOCX)

S2 Table Results of logistic regression predicting statistical significance of rank-correlation test with discipline and control variable number of effect sizes in a subset. (DOCX)

S3 Table Results of logistic regression predicting statistical significance of *p-*uniform’s publication bias test with discipline and control variable number of statistically significant effect sizes in a subset. (DOCX)

S4 Table Results of logistic regression predicting statistical significance of test of excess significance with discipline and control variable number of effect sizes in a subset. (DOCX)

S5 Table Results of multilevel logistic regression predicting statistical significance of Egger’s regression test with discipline and control variable number of effect sizes in a subset. (DOCX)

S6 Table Results of multilevel logistic regression predicting statistical significance of rank-correlation test with discipline and control variable number of effect sizes in a subset. (DOCX)

S7 Table Results of multilevel logistic regression predicting statistical significance of *p-*uniform’s publication bias test with discipline and control variable number of statistically significant effect sizes in a subset. (DOCX)

S8 Table Results of multilevel logistic regression predicting statistical significance of test of excess significance with discipline and control variable number of effect sizes in a subset. (DOCX)

S9 Table Results of meta-meta regression with a random effect to take into account that the subsets were nested in meta-analyses. The dependent variable is the absolute value of the random-effects meta-analysis effect size estimate with predictors discipline, *I*^2^-statistic, harmonic mean of the standard error (standard error), proportion of statistically significant effect sizes in a subset (Prop. sig. effect sizes), and number of effect sizes in a subset.
(DOCX)

S10 Table Results of meta-meta regression with a random effect to take into account that the subsets were nested in meta-analyses. The dependent variable is the absolute value of *p-*uniform’s effect size estimate with predictors discipline, *I*^2^-statistic, harmonic mean of the standard error (standard error), proportion of statistically significant effect sizes in a subset (Prop. sig. effect sizes), and number of effect sizes in a subset.
(DOCX)

S11 Table Results of quantile regression with the median of *p-*uniform’s effect size estimates and predictors discipline, *I*^2^-statistic, harmonic mean of the standard error (standard error), proportion of statistically significant effect sizes in a subset (Prop. sig. effect sizes), and number of effect sizes in a subset. (DOCX)

S12 Table Results of meta-meta-regression with a random effect to take into account that the subsets were nested in meta-analyses. The dependent variable is the effect size overestimation in random-effects meta-analysis when compared to *p-*uniform (*Y*) and predictors discipline, *I*^2^-statistic, harmonic mean of the standard error (standard error), proportion of statistically significant effect sizes in a subset (Prop. sig. effect sizes), and number of effect sizes in a subset.
(DOCX)

S13 Table Results of quantile regression with the median of effect size overestimation in random-effects meta-analysis when compared to *p-*uniform (*Y*) and predictors discipline, *I*^2^-statistic, harmonic mean of the standard error (standard error), proportion of statistically significant effect sizes in a subset (Prop. sig. effect sizes), and number of effect sizes in a subset. (DOCX)

S1 File List of references of meta-analyses where the data of the primary studies were obtained after contacting the corresponding author. (DOCX)
