# Supplementary material for: Publication bias examined in meta-analyses from psychology and medicine: A meta-meta-analysis
Source: PLoS One. 2019 Apr 12;14(4):e0215052. doi: 10.1371/journal.pone.0215052 (PMC6461282; doi:10.1371/journal.pone.0215052)
Supplement: S2 Table — (DOCX) [file pone.0215052.s002.docx]

|  | B (SE) | *z-*value (*p*-value) | OR | 95% CI for OR |
| --- | --- | --- | --- | --- |
| Intercept | -2.603 (0.209) | -12.456 (< .001) | 0.074 | 0.048;0.11 |
| Discipline | 0.4 (0.248) | 1.613 (.054) | 1.491 | 0.921;2.441 |
| Number of effect sizes | 0.029 (0.011) | 2.557 (.011) | 1.029 | 1.007;1.054 |

*Note.* CDSR is the reference category for discipline. *p-*values for the intercept and number of effect are two-tailed whereas the *p*-value for discipline is one-tailed. OR = odds ratio. CI = profile likelihood confidence interval
