# Supplementary material for: Publication bias examined in meta-analyses from psychology and medicine: A meta-meta-analysis
Source: PLoS One. 2019 Apr 12;14(4):e0215052. doi: 10.1371/journal.pone.0215052 (PMC6461282; doi:10.1371/journal.pone.0215052)
Supplement: S5 Table — (DOCX) [file pone.0215052.s005.docx]

|  | B (SE) | *z-*value (*p*-value) | OR | 95% CI for OR |
| --- | --- | --- | --- | --- |
| Intercept | -2.389 (0.256) | -9.331 (<.001) | 0.092 | 0.052;0.144 |
| Discipline | -0.025 (0.287) | -0.087 (.465) | 0.975 | 0.547;1.732 |
| Number of effect sizes | 0.035 (0.013) | 2.605 (.009) | 1.036 | 1.009;1.065 |

*Note.* CDSR is the reference category for discipline. *p-*values for the intercept and number of effect sizes are two-tailed whereas the *p*-value for discipline is one-tailed. OR = odds ratio. CI = profile likelihood confidence interval. Conditional intraclass correlation = 16.3%.
