# Supplementary material for: Publication bias examined in meta-analyses from psychology and medicine: A meta-meta-analysis
Source: PLoS One. 2019 Apr 12;14(4):e0215052. doi: 10.1371/journal.pone.0215052 (PMC6461282; doi:10.1371/journal.pone.0215052)
Supplement: S6 Table — (DOCX) [file pone.0215052.s006.docx]

|  | B (SE) | *z-*value (*p*-value) | OR | 95% CI for OR |
| --- | --- | --- | --- | --- |
| Intercept | -2.942 (0.31) | -9.479 (0) | 0.053 | 0.026;0.091 |
| Discipline | 0.283 (0.331) | 0.854 (0.1965) | 1.327 | 0.681;2.581 |
| Number of effect sizes | 0.034 (0.015) | 2.305 (0.021) | 1.035 | 1.006;1.068 |

*Note.* CDSR is the reference category for discipline. *p-*values for the intercept and number of effect sizes are two-tailed whereas the *p*-value for discipline is one-tailed. OR = odds ratio. CI = profile likelihood confidence interval. Conditional intraclass correlation = 22.3%.
