# Supplementary material for: Publication bias examined in meta-analyses from psychology and medicine: A meta-meta-analysis
Source: PLoS One. 2019 Apr 12;14(4):e0215052. doi: 10.1371/journal.pone.0215052 (PMC6461282; doi:10.1371/journal.pone.0215052)
Supplement: S7 Table — (DOCX) [file pone.0215052.s007.docx]

|  | B (SE) | *z-*value (*p*-value) | OR | 95% CI for OR |
| --- | --- | --- | --- | --- |
| Intercept | -2.616 (0.332) | -7.881 (<.001) | 0.073 | 0.036;0.136 |
| Discipline | 0.8 (0.361) | 2.217 (0.014) | 2.226 | 1.123;4.675 |
| Number of effect sizes | -0.067 (0.066) | -1.019 (.308) | 0.935 | 0.807;1.042 |

*Note.* CDSR is the reference category for discipline. *p-*values for the intercept and number of effect sizes are two-tailed whereas the *p*-value for discipline is one-tailed. OR = odds ratio. CI = profile likelihood confidence interval. Conditioanl intraclass correlation = 0%.
