# Supplementary material for: Publication bias examined in meta-analyses from psychology and medicine: A meta-meta-analysis
Source: PLoS One. 2019 Apr 12;14(4):e0215052. doi: 10.1371/journal.pone.0215052 (PMC6461282; doi:10.1371/journal.pone.0215052)
Supplement: S8 Table — (DOCX) [file pone.0215052.s008.docx]

|  | B (SE) | *z-*value (*p*-value) | OR | 95% CI for OR |
| --- | --- | --- | --- | --- |
| Intercept | -3.403 (0.278) | -12.22 (<.001) | 0.033 | 0.019;0.056 |
| Discipline | 0.295 (0.339) | 0.871 (0.192) | 1.344 | 0.694;2.652 |
| Number of effect sizes | 0.04 (0.013) | 3.195 (.001) | 1.041 | 1.016;1.068 |

*Note.* CDSR is the reference category for discipline. *p-*values for the intercept and number of effect sizes are two-tailed whereas the *p*-value for discipline is one-tailed. OR = odds ratio. CI = profile likelihood confidence interval. Conditional intraclass correlation = 0%.
