# Supplementary material for: Publication bias examined in meta-analyses from psychology and medicine: A meta-meta-analysis
Source: PLoS One. 2019 Apr 12;14(4):e0215052. doi: 10.1371/journal.pone.0215052 (PMC6461282; doi:10.1371/journal.pone.0215052)
Supplement: S9 Table — The dependent variable is the absolute value of the random-effects meta-analysis effect size estimate with predictors discipline, I2-statistic, harmonic mean of the standard error (standard error), proportion of statistically significant effect sizes in a subset (Prop. sig. effect sizes), and number of effect sizes in a subset. (DOCX) [file pone.0215052.s009.docx]

|  | B (SE) | *t-*value (*p*-value) | 95% CI |
| --- | --- | --- | --- |
| Intercept | 0.075 (0.029) | 2.576 (.010) | 0.018;0.132 |
| Discipline | 0.115 (0.030) | 3.828 (<.001) | 0.056;0.174 |
| *I*^2^-statistic | 0.001 (0.0004) | 3.955 (<.001) | 0.001;0.002 |
| Standard error | 0.642 (0.082) | 7.786 (<.001) | 0.481;0.804 |
| Number of effect sizes | -0.001 (0.001) | -1.575 (0.058) | -0.002;0.0002 |

*Note.* CDSR is the reference category for discipline. *p-*values for discipline and harmonic mean of the standard error are one-tailed whereas the other *p-*values are two-tailed. CI = Wald-based confidence interval. Conditional intraclass correlation = 0.9%.
