# Supplementary material for: Publication bias examined in meta-analyses from psychology and medicine: A meta-meta-analysis
Source: PLoS One. 2019 Apr 12;14(4):e0215052. doi: 10.1371/journal.pone.0215052 (PMC6461282; doi:10.1371/journal.pone.0215052)
Supplement: S10 Table — The dependent variable is the absolute value of p-uniform’s effect size estimate with predictors discipline, I2-statistic, harmonic mean of the standard error (standard error), proportion of statistically significant effect sizes in a subset (Prop. sig. effect sizes), and number of effect sizes in a subset. (DOCX) [file pone.0215052.s010.docx]

|  | B (SE) | *t-*value (*p*-value) | 95% CI |
| --- | --- | --- | --- |
| Intercept | 0.77 (0.689) | 1.118 (.264) | -0.58;2.121 |
| Discipline | 0.001 (0.497) | 0.001 (.999) | -0.973;0.974 |
| *I*^2^-statistic | 0.013 (0.014) | 0.939 (.174) | -0.014;0.039 |
| Standard error | 3.767 (2.587) | 1.456 (.146) | -1.303;8.838 |
| Prop. sig. effect sizes | -1.287 (0.797) | -1.615 (.107) | -2.849;0.275 |
| Number of effect sizes | -0.02 (0.015) | -1.363 (.173) | -0.049;0.009 |

*Note.* CDSR is the reference category for discipline. *p-*value for the *I*^2^-statistic is one-tailed whereas the other *p-*values are two-tailed. CI = Wald-based confidence interval. Conditional intraclass correlation = 0%.
