# Supplementary material for: Publication bias examined in meta-analyses from psychology and medicine: A meta-meta-analysis
Source: PLoS One. 2019 Apr 12;14(4):e0215052. doi: 10.1371/journal.pone.0215052 (PMC6461282; doi:10.1371/journal.pone.0215052)
Supplement: S11 Table — (DOCX) [file pone.0215052.s011.docx]

|  | B (SE) | *t-*value (*p*-value) | 95% CI |
| --- | --- | --- | --- |
| Intercept | -0.067 (0.071) | -0.956 (.34) | -0.192;-0.046 |
| Discipline | 0.009 (0.041) | 0.224 (.823) | -0.038;0.084 |
| *I*^2^-statistic | -0.001 (0.001) | -0.894 (.814) | -0.003;0.001 |
| Standard error | 2.021 (0.254) | 7.969 (<.001) | 1.725;2.283 |
| Prop. sig. effect sizes | 0.196 (0.083) | 2.353 (.019) | 0.085;0.339 |
| Number of effect sizes | -0.002 (0.002) | -0.86 (.39) | -0.007;0.0002 |

*Note.* CDSR is the reference category for discipline. *p-*value for the *I*^2^-statistic is one-tailed whereas the other *p-*values are two-tailed. CI = confidence interval based on inverting a rank test.
