# Supplementary material for: Publication bias examined in meta-analyses from psychology and medicine: A meta-meta-analysis
Source: PLoS One. 2019 Apr 12;14(4):e0215052. doi: 10.1371/journal.pone.0215052 (PMC6461282; doi:10.1371/journal.pone.0215052)
Supplement: S12 Table — The dependent variable is the effect size overestimation in random-effects meta-analysis when compared to p-uniform (Y) and predictors discipline, I2-statistic, harmonic mean of the standard error (standard error), proportion of statistically significant effect sizes in a subset (Prop. sig. effect sizes), and number of effect sizes in a subset. (DOCX) [file pone.0215052.s012.docx]

|  | B (SE) | *t-*value (*p*-value) | 95% CI |
| --- | --- | --- | --- |
| Intercept | -0.017 (0.033) | -0.517 (.605) | -0.083;0.048 |
| Discipline | -0.04 (0.024) | -1.651 (.951) | -0.087;0.007 |
| *I*^2^-statistic | -0.004 (0.001) | -5.338 (<.001) | -0.005;-0.002 |
| Standard error | 0.172 (0.126) | 1.371 (.086) | -0.074;0.418 |
| Prop. sig. effect sizes | 0.182 (0.039) | 4.713 (<.001) | 0.106;0.258 |
| Number of effect sizes | -0.001 (0.001) | -2.064 (.04) | -0.003;-0.0001 |

*Note.* CDSR is the reference category for discipline. *p-*values for discipline, the *I*^2^-statistic, and the harmonic mean of the standard error are one-tailed whereas the other *p-*values are two-tailed. CI = Wald-based confidence interval. Conditional intraclass correlation = 0%
