# Supplementary material for: Publication bias examined in meta-analyses from psychology and medicine: A meta-meta-analysis
Source: PLoS One. 2019 Apr 12;14(4):e0215052. doi: 10.1371/journal.pone.0215052 (PMC6461282; doi:10.1371/journal.pone.0215052)
Supplement: S13 Table — (DOCX) [file pone.0215052.s013.docx]

|  | B (SE) | *t-*value (*p*-value) | 95% CI |
| --- | --- | --- | --- |
| Intercept | -0.007 (0.046) | -0.148 (.883) | -0.014;0.055 |
| Discipline | -0.003 (0.016) | -0.2 (.579) | -0.02;0.021 |
| *I*^2^-statistic | -0.0003 (0.0004) | -0.745 (.229) | -0.001;-0.0001 |
| Standard error | 0.279 (0.148) | 1.889 (.03) | 0.182;0.386 |
| Prop. sig. effect sizes | -0.002 (0.002) | -1.53 (.127) | -0.004;0.001 |
| Number of effect sizes | 0.017 (0.035) | 0.487 (.627) | -0.011;0.052 |

*Note.* CDSR is the reference category for discipline. *p-*values for discipline, the *I*^2^-statistic, and the harmonic mean of the standard error are one-tailed whereas the other *p-*values are two-tailed. CI = confidence interval based on inverting a rank test.
