# Supplementary material for: Publication bias examined in meta-analyses from psychology and medicine: A meta-meta-analysis
Source: PLoS One. 2019 Apr 12;14(4):e0215052. doi: 10.1371/journal.pone.0215052 (PMC6461282; doi:10.1371/journal.pone.0215052)
Supplement: S1 File — (DOCX) [file pone.0215052.s014.docx]

**Supporting Information 1 Text of van Aert, Wicherts, van Assen (2018)**

List of references of meta-analyses where the data of the primary studies were obtained after contacting the corresponding author:

1. Richler JJ, Gauthier I. A meta-analysis and review of holistic face processing. Psychological Bulletin. 2014;140(5):1281-302. doi: 10.1037/a0037004.

2. Trahan LH, Stuebing KK, Fletcher JM, Hiscock M. The Flynn effect: A meta-analysis. Psychological Bulletin. 2014;140(5):1332-60. doi: 10.1037/a0037173.

3. Schmitt MT, Branscombe NR, Postmes T, Garcia A. The consequences of perceived discrimination for psychological well-being: A meta-analytic review. Psychological Bulletin. 2014;140(4):921-48. doi: 10.1037/a0035754.

4. Murayama K, Miyatsu T, Buchli D, Storm BC. Forgetting as a consequence of retrieval: A meta-analytic review of retrieval-induced forgetting. Psychological Bulletin. 2014;140(5):1383-409. doi: 10.1037/a0037505.

5. Snyder HR. Major depressive disorder is associated with broad impairments on neuropsychological measures of executive function: A meta-analysis and review. Psychological Bulletin. 2013;139(1):81-132. doi: 10.1037/a0028727.

6. Murayama K, Elliot AJ. The competition–performance relation: A meta-analytic review and test of the opposing processes model of competition and performance. Psychological Bulletin. 2012;138(6):1035-70. doi: 10.1037/a0028324.

7. Rhodes MG, Anastasi JS. The own-age bias in face recognition: A meta-analytic and theoretical review. Psychological Bulletin. 2012;138(1):146-74. doi: 10.1037/a0025750.

8. Lench HC, Flores SA, Bench SW. Discrete emotions predict changes in cognition, judgment, experience, behavior, and physiology: A meta-analysis of experimental emotion elicitations. Psychological Bulletin. 2011;137(5):834-55. doi: 10.1037/a0024244.

9. Haedt-Matt AA, Keel PK. Revisiting the affect regulation model of binge eating: A meta-analysis of studies using ecological momentary assessment. Psychological Bulletin. 2011;137(4):660-81. doi: 10.1037/a0023660.

10. Anderson CA, Shibuya A, Ihori N, Swing EL, Bushman BJ, Sakamoto A, et al. Violent video game effects on aggression, empathy, and prosocial behavior in Eastern and Western countries: A meta-analytic review. Psychological Bulletin. 2010;136(2):151-73. doi: 10.1037/a0018251.

11. Moskowitz JT, Hult JR, Bussolari C, Acree M. What works in coping with HIV? A meta-analysis with implications for coping with serious illness. Psychological Bulletin. 2009;135(1):121-41. doi: 10.1037/a0014210.
